# Visualizing fatigue mechanisms in non-communicable diseases: an integrative approach with multi-omics and machine learning

**DOI:** 10.1186/s12911-025-03034-3

**Published:** 2025-06-03

**Authors:** Yusuke Kobayashi, Naoki Fujiwara, Yuki Murakami, Shoichi Ishida, Sho Kinguchi, Tatsuya Haze, Kengo Azushima, Akira Fujiwara, Hiromichi Wakui, Masayoshi Sakakura, Kei Terayama, Nobuhito Hirawa, Tetsuo Isozaki, Hiroaki Yasuzaki, Hajime Takase, Yuichiro Yano, Kouichi Tamura

**Affiliations:** 1 YCU Co-Creation Innovation Center, Yokohama City University, Yokohama, Japan; 2 Center for Novel and Exploratory Clinical Trials (Y-NEXT), Yokohama City University Hospital, Yokohama, Japan; 3 Department of Medical Science and Cardiorenal Medicine, Yokohama City University Graduate School of Medicine, Yokohama, Japan; 4 Graduate School of Medical Life Science, Yokohama City University, Yokohama, Japan; 5 Department of Nephrology and Hypertension, Yokohama City University Medical Center, Yokohama, Japan; 6 Koiso Medical Clinic, Yokosuka, Japan; 7 Kotani Medical Clinic, Yokohama, Japan; 8 Department of General Medicine, Juntendo University Faculty of Medicine, Tokyo, Japan; 9 92-2-1, Minatomirai, Nishi, Yokohama, Kanagawa, 220-8107 Japan

**Keywords:** Fatigue, Chronic disease, Multi-Omics, Machine learning

## Abstract

**Background:**

Fatigue is a prevalent and debilitating symptom of non-communicable diseases (NCDs); however, its biological basis are not well-defined. This exploratory study aimed to identify key biological drivers of fatigue by integrating metabolomic, microbiome, and genetic data from blood and saliva samples using a multi-omics approach.

**Methods:**

Metabolomic, microbiome, and single nucleotide polymorphisim analyses were conducted on saliva and blood samples from 52 patients with NCDs. Fatigue dimensions were assessed using the Multidimensional Fatigue Inventory and correlated with biological markers. LightGBM, a gradient boosting algorithm, was used for fatigue prediction, and model performance was evaluated using the F1-score, accuracy, and receiver operating characteristic area under the curve using leave-one-out cross-validation. Statistical analyses included correlation tests and multiple comparison adjustments (*p* < 0.05; false discovery rate <0.05). This study was approved by the Yokohama City University Hospital Ethics Committee (F230100022).

**Results:**

Plasmalogen synthesis was significantly associated with physical fatigue in both blood and saliva samples. Additionally, homocysteine degradation and catecholamine biosynthesis in the blood were significantly associated with mental fatigue (Holm *p* < 0.05). Microbial imbalances, including reduced levels of *Firmicutes negativicutes* and *Patescibacteria saccharimonadia*, correlated with general and physical fatigue (*r* = − 0.379, *p* = 0.006). Genetic variants in genes, such as *GPR180, NOTCH3, SVIL, HSD17B11*, and *PLXNA1*, were linked to various fatigue dimensions (r range: −0.539–0.517, *p* < 0.05). Machine learning models based on blood and salivary biomarkers achieved an F1-score of approximately 0.7 in predicting fatigue dimensions.

**Conclusion:**

This study provides preliminary insights into the potential involvement of alterations in lipid metabolism, catecholamine biosynthesis disruptions, microbial imbalances, and specific genetic variants in fatigue in patients with NCDs. These findings lay the groundwork for personalized interventions, although further validation and model refinement across diverse populations are needed to enhance the prediction performance and clinical applicability.

**Supplementary Information:**

The online version contains supplementary material available at 10.1186/s12911-025-03034-3.

## Novel insights from the study

1. Lipid metabolism has emerged as a key contributor to general and physical fatigue, specifically through the biosynthesis of unsaturated fatty acids and plasmalogens, synthesis.
2. Microbial dysbiosis in the salivary microbiota, particularly the depletion of *Firmicutes negativicutes* and *Patescibacteria saccharimonadia*, significantly correlated with increased fatigue.
3. Genetic variations, particularly in *GPR180, NOTCH3, SVIL, HSD17B11*, and *PLXNA1* are associated with multiple dimensions of fatigue, emphasizing the role of genetic predisposition across various fatigue factors.
4. Blood- and salivary-based biomarkers demonstrated potential for fatigue prediction, with machine learning models showing an F1-score of approximately 0.7 for predicting fatigue dimensions.

## Introduction

Fatigue is a common and debilitating symptom of non-communicable diseases (NCDs) [1] such as hypertension, dyslipidemia, diabetes, and chronic kidney disease (CKD). It significantly impairs quality of life [2, 3] and remains a major concern in patients with chronic conditions. Although fatigue is a shared experience across many chronic conditions, its underlying biological mechanisms vary, depending on the disease type. In NCDs, fatigue has been linked to disruptions in metabolic pathways, neurotransmitter signaling, and immune regulation; however, its precise contributors remain poorly understood [4, 5, 6]. Studies have suggested that metabolic, microbial, and genetic factors contribute to fatigue [6, 7, 8], although their relevance may vary, depending on the condition. For example, a study on chronic fatigue syndrome (CFS) indicates that dysregulated lipid metabolism, reduced butyrate production, and gut dysbiosis may contribute to the severity of fatigue. [7] Similarly, altered glucose metabolism and insulin resistance are associated with fatigue in patients with type 2 diabetes, highlighting the role of metabolic imbalances [6]. Additionally, genetic variations affecting mitochondrial function, neurotransmitter signaling, and immune response have been implicated in fatigue susceptibility [8]. These findings underscore the need to investigate fatigue as a multifactorial condition influenced by the complex interplay between metabolism, the microbiome, and genetic factors.

Previous studies have largely focused on individual metabolic, microbial, or genetic factors, limiting a comprehensive understanding of fatigue mechanisms [7, 8] Given the multifactorial nature of fatigue mechanisms, a systems-level approach is needed to evaluate the complex interactions between these biological components [1, 2, 8].

Currently, fatigue assessment relies primarily on self-reported measures that capture subjective experiences but lacks standardized objective biomarkers [1, 5]. Identifying biomarkers associated with fatigue could complement these assessments by providing physiological correlates that can improve diagnosis, treatment monitoring, and the development of personalized therapeutic strategies.

In addition to its direct impact on daily functioning, fatigue in patients with NCDs may serve as an early indicator of disease progression, emphasizing its potential as a predictive marker [9]. In addition to individual health consequences, fatigue significantly affects productivity, contributing to presenteeism, absenteeism, and economic burden [10, 11, 12]. Moreover, the growing recognition of post-COVID-19 fatigue highlights the need for improved fatigue management for various health conditions [13]. Recently, various machine learning methods have been developed, and these approaches have the potential to contribute to predicting fatigue using biomarkers [14, 15, 16].

This study aimed to bridge the existing gaps in fatigue studies through a multi-omics approach by integrating metabolomic, microbiome, and genetic data from blood and saliva samples. Additionally, we used machine learning techniques to explore potential biomarkers and predictive models of fatigue in patients with NCDs. By leveraging these biological datasets, we aimed to provide preliminary insights into the mechanisms underlying fatigue and assess the potential of biomarker-based fatigue classification. This exploratory study lays the foundation for future studies aimed at improving the diagnosis of fatigue, tracking disease progression, and providing targeted interventions. Furthermore, understanding the biological basis of fatigue may offer insights into its role as a signal for declining health status, supporting early interventions to enhance patient well-being.

## Methods

### Participants

This study recruited patients with NCDs, including hypertension, diabetes mellitus, dyslipidemia, and CKD, from the Kotani Medical Clinic and Koiso Medical Clinic. Eligibility was assessed according to inclusion and exclusion criteria.

### Inclusion and exclusion criteria

Patients were enrolled if they met the inclusion criteria and did not meet any exclusion criteria. Participants were eligible for inclusion if they were adults (aged ≥ 18 years old) diagnosed with at least one of the following NCDs for at least six months, were receiving pharmacological treatment, or had a physician-confirmed diagnosis:

- Hypertension: systolic blood pressure ≥ 140 mmHg or diastolic blood pressure ≥ 90 mmHg.
- Diabetes: fasting plasma glucose ≥ 126 mg/dL or HbA1c ≥ 6.5%.
- Dyslipidemia: low-density lipoprotein cholesterol ≥ 140 mg/dL, high-density lipoproteitn cholesterol < 40 mg/dL, or triglycerides ≥ 150 mg/dL.
- CKD: estimated glomerular filtration rate < 60 mL/min/1.73 or urinary albumin-to-creatinine ratio ≥ 30 mg/gCr.

Eligible patients underwent routine blood tests on the day of recruitment. All the participants were given a comprehensive explanation of the study and provided written informed consent before participation. Broad inclusion criteria were applied to enhance the generalizability of the results.

Exclusion criteria included patients with severe oral dryness, which could interfere with saliva collection and individuals with a body temperature >  37.5 °C. Patients unable to provide sufficient saliva were excluded because of the necessity of this sample type for metabolite analysis, and individuals with temporary infections were excluded to reduce confounding factors.

### Participant recruitment and ethical approval

Eligible participants were identified through electronic medical records and approached by clinic staff. Patients who met the inclusion criteria and agreed to participate provided written informed consent. This prospective cross-sectional study was approved by the Yokohama City University Hospital Ethics Committee (approval code: F230100022) and conducted in compliance with the Declaration of Helsinki.

### Patient characteristics

Demographic and clinical data, including age, sex, medical history, and current medications, were collected from all the participants to comprehensively characterize the study population.

### Evaluation of fatigue

Fatigue was evaluated using the Multidimensional Fatigue Inventory (MFI) [17, 18], which is a 20-item questionnaire assessing five fatigue dimensions: general fatigue, physical fatigue, mental fatigue, reduced activity, and reduced motivation. Physical fatigue refers to a subjective sense of physical exhaustion or tiredness, affecting one’s ability to exert physical energy, whereas reduced activity denotes a behavioral outcome characterized by a diminished frequency or intensity of activity due to fatigue. Similarly, mental fatigue describes cognitive exhaustion, affecting concentration and mental stamina, whereas reduced motivation reflects a decline in the willingness or desire to engage in tasks, likely due to sustained fatigue [17, 18]. Each dimension was analyzed in relation to patient characteristics, metabolomic profiles, microbiota, and genetic variants. Additionally, machine learning models were developed to predict outcomes across these fatigue dimensions.

### Biological sample collection

Blood, saliva, and buccal mucosal cell samples were collected from all the participants. Blood samples were obtained by venipuncture using a VP-NA070K kit (Terumo Corporation, Tokyo, Japan), centrifuged to isolate the serum, and stored at −20 °C for metabolomic analysis. Saliva samples collected using Falcon 50 mL Conical Centrifuge Tube (Corning Inc., Corning, NY, USA) were likewise frozen at −20 °C and utilized for metabolomic and 16S rRNA analyses. Buccal mucosal cells were collected via oral swabbing, stored in iSWAB DNA Collection Tubes (Mawi DNA Technologies, Pleasanton, CA, USA), and later used for whole exome sequencing.

### Metabolomic analysis using gas chromatography–mass spectrometry (GC–MS)

Centrifuged saliva and serum samples were used for metabolite extraction, with 50 µL of supernatant taken for analysis. The samples were derivatized using methoximation and trimethylsilylation and subsequently analyzed using a GCMS-TQ8030 GC–MS (Shimadzu Corporation, Kyoto, Japan) to quantify metabolites, including organic acids, sugars, and amino acids. Detailed preprocessing and analytical conditions have been described previously [19]. The metabolites were quantified by normalizing peak areas to an internal standard (2-isopropylmalic acid). Quality control (QC) samples were analyzed at regular intervals to ensure analytical consistency, and metabolites with coefficients of variation >  25% in the QC samples were excluded from the final dataset.

### Enrichment analysis and pathway analysis of metabolome

Serum and saliva metabolomic data were analyzed using MetaboAnalyst 6.0 (McGill University, Montreal, Canada). The pathway analysis module facilitates both pathway enrichment and topology analyses. Initially, metabolite concentration data were formatted.csv files, with samples in rows, compounds in columns, and phenotype labels to accommodate binary, multi-group, or continuous analyses. The compound labels were standardized for accurate alignment with the Kyoto Encyclopedia of Genes and Genomes pathway library for Homo sapiens. Pathway enrichment analysis was conducted using GlobalTest to identify pathways with significant alterations. Pathway topology analysis used relative betweenness centrality to assess node importance, allowing the integration of structural information to identify key affected pathways.

### 16S rRNA analysis

Saliva samples were subjected to 16S rRNA gene sequencing to characterize microbial communities. Extraction of DNA was performed using a Promega Maxwell RSC Instrument (Promega Corporation, Madison, WI, USA), and the V3–V4 regions of the 16S rRNA gene were amplified using universal primers. Sequencing was performed on HiSeq 2500 or NovaSeq 6000 platforms (Illumina, San Diego, CA, USA). Sequences were aligned with known 16S rRNA gene databases to identify bacterial taxa and provide a detailed bacterial community profile. Sequences of DNA with high similarity were grouped and bacterial proportions were calculated based on sequence counts.

Pearson’s correlation analyses were performed to explore the relationship between fatigue factors and microbial diversity. Alpha diversity was assessed using the Shannon and Simpson indices. Beta diversity was calculated using the Bray–Curtis dissimilarity index and visualized via multidimensional scaling (MDS). The first two dimensions from the MDS (Dim1 and Dim2) were used to represent the between-sample variability in the microbial composition. Additionally, the Pearson’s correlation analyses were used to examine the association between individual microbial taxa and fatigue factors.

### Whole exome sequencing

Exraction of DNA was performed using a Promega Maxwell RSC Instrument (Promega Corporation, Madison, WI, USA). The extraction process was performed using a Maxwell RSC Blood DNA Kit (AS1400) (Promega Corporation, Madison, WI, USA). We used human buccal samples for volume of 200.0 µL for the extraction. Elution step was performed with 50.0 µL of elution buffer. Whole exome sequencing was performed using the HiSeq 2500 or NovaSeq 6000 sequencing platforms (Illumina, San Diego, CA, USA) to generate high-throughput sequencing data. Sequencing was conducted according to the manufacturer’s protocol to ensure optimal coverage and depth across the targeted exonic regions for variant detection and analysis. Sequencing data were analyzed to identify genetic variants associated with the studied conditions.

### Development of machine learning-based models for predicting fatigue

Prediction models for physical and mental fatigue were developed using LightGBM [20], a gradient-boosting decision-tree algorithm. The dataset comprised 52 patients with NCDs. Three sets of explanatory variables were used to build the prediction models: (1) salivary biomarkers (112 variables); (2) blood biomarkers (112 variables); and (3) a combined dataset (265 variables) that included salivary and blood biomarkers along with additional variables such as age, NCD category, and microbiome data. All explanatory variables were standardized.

Fatigue levels were categorized on the basis of the Japanese version of the MFI [18]. For physical fatigue, patients with an MFI physical fatigue score of ≤  10.7 (mean score) were classified as “no fatigue” (*n* = 22), while scores > 10.7 indicated “fatigue present” (*n* = 30). Similarly, mental fatigue was classified, with scores ≤  10.6 as “no fatigue” (*n* = 22) and >  10.6 as “fatigue present” (*n* = 30).

To mitigate risks of overfitting and ensure robust model evaluation, we employed leave-one-out cross-validation for prediction model evaluation.

Model performance was evaluated using the F1 score, accuracy, and receiver operating characteristic area under the curve (ROC-AUC). The LightGBM hyperparameters were set to default values, and feature selection was not implemented. Model construction and validation were conducted using Python 3.9.0, scikit-learn 1.5.0, pandas 2.2.2, numpy 1.26.4, shap 0.45.1, and LightGBM 4.3.0 libraries.

### Statistical analysis

All statistical analyses were conducted using SPSS software version 29.0.0.0 (IBM Corp., Armonk, NY, USA). Descriptive statistics, including means, standard deviations, medians, and interquartile ranges (IQR), were used to summarize the participant characteristics and study data. The Pearson’s correlation analysis was used to explore the associations among fatigue dimensions, microbiome profiles, metabolomic profiles, and genetic data. For pathway enrichment analyses, Holm-adjusted *p*-values and false discovery rates (FDR) were calculated to control for multiple comparisons, with significance thresholds set at *p* < 0.05 and FDR < 0.05. Leave-one-out cross validation was applied to evaluate the robustness of the machine learning models. Additional statistical tests, including the t-tests, chi-square tests, and Mann–Whitney U tests, were applied as appropriate to identify significant relationships within the dataset and to account for the distributional characteristics of the data.

## Results

### Participant characteristics

Baseline characteristics of the study participants are summarized in Table 1. The participants had a mean age of 67.9 ± 11.2 years, and 55.8% were males. Mean body mass index (BMI) was 24.5 ± 3.3 kg/m². Among the participants, 63.5% had a history of hypertension, 75.0% had dyslipidemia, 25.0% had diabetes, and 38.5% had CKD. Mean systolic and diastolic blood pressures were 129.3 ± 14.1 and 74.6 ± 12.7 mmHg, respectively. Hemoglobin and albumin levels averaged 14.0 ± 2.1 and 4.3 ± 0.3 g/dL, respectively, reflecting generally stable health within the cohort.

**Table 1:** Characteristics of participants with non-communicable diseases

	Participants with NCDs (*n* = 52) Mean (SD) or N (%)
Age, years	67.9 (11.2)
Sex, male (%)	29 (55.8)
Body mass index, kg/m ^2^	24.5 (3.3)
History of hypertension, *n*(%)	33 (63.5)
History of dyslipidemia, *n*(%)	39 (75.0)
History of diabetes, *n*(%)	13 (25.0)
History of chronic kidney disease, *n*(%)	20 (38.5)
Systolic blood pressure, mmHg	129.3 (14.1)
Diastolic blood pressure, mmHg	74.6 (12.7)
Heart rate, bpm	75.8 (10.3)
Hemoglobin, g/dL	14.0 (2.1)
Albumin, g/dL	4.3 (0.3)
AST, U/L	25.5 (8.4)
ALT, U/L	24.9 (14.3)
C-reactive protein, mg/dL	0.24 (0.24)
e-GFR, ml/min	65.7 (17.2)
Uric acid, mg/dL	5.5 (1.4)
Sodium, mEq/L	141.1 (2.4)
Potassium, mEq/L	4.0 (0.3)
Chloride, mEq/L	103.9 (2.4)
Phosphate, mg/dL	3.6 (0.3)
Fasting glucose, mg/dL	118.9 (46.0)
LDL cholesterol, mg/dL	109.9 (22.5)
HDL cholesterol, mg/dL	59.8 (18.2)
Triglyceride, mg/dL	160.5 (83.1)
Use of anti-hypertensives, *n*(%)	33 (63.5)
Use of anti-dyslipidemia, *n*(%)	30 (57.7)
Use of anti-diabetic, *n*(%)	12 (23.1)

Demographic and clinical characteristics of participants with NCDs (*n* = 52). Data are presented as mean (SD) for continuous variables and as counts (percentages) for categorical variables. Abbreviations: NCDs, Non-Communicable Diseases; BMI, Body Mass Index; AST, Aspartate Aminotransferase; ALT, Alanine Aminotransferase; CRP, C-reactive Protein; e-GFR, estimated Glomerular Filtration Rate; LDL, Low-Density Lipoprotein; HDL, High-Density Lipoprotein

### Fatigue in patients with NCDs

Table 2 displays fatigue levels, assessed using the MFI, among the participants. Median general fatigue score was 10.0 (IQR: 5.8). Physical fatigue had a median score of 11.5 (IQR: 5.0). Mental fatigue had a median score of 11.0 (IQR: 4.0). Reduced activity and reduced motivation had median scores of 9.0 (IQR: 4.0) and 10.0 (IQR: 4.0), respectively. Total MFI score, reflecting overall fatigue burden, had a median of 53.5 (IQR: 21.3), indicating notable variability in fatigue severity across the participants.

**Table 2:** Characteristics of fatigue in participants with non-communicable diseases

	Participants with NCDs (*n* = 52) Median (IQR)
General fatigue	10.0 (5.8)
Physical fatigue	11.5 (5.0)
Mental fatigue	11.0 (4.0)
Reduced activity	9.0 (4.0)
Reduced motivation	10.0 (4.0)
Total MFI score	53.5 (21.3)

Distribution of fatigue scores for each dimension among participants with NCDs (*n* = 52). Fatigue scores are expressed as median (IQR) for general fatigue, physical fatigue, mental fatigue, reduced activity, reduced motivation, and total MFI score. *Abbreviations* NCDs, Non-Communicable Diseases; MFI, Multidimensional Fatigue Inventory

### Metabolomics and fatigue

Blood and salivary metabolomic data were analyzed using MetaboAnalyst 6.0 (McGill University, Montreal, Canada) in conjunction with metabolite quantification using GC–MS.

**Fig. 1:**
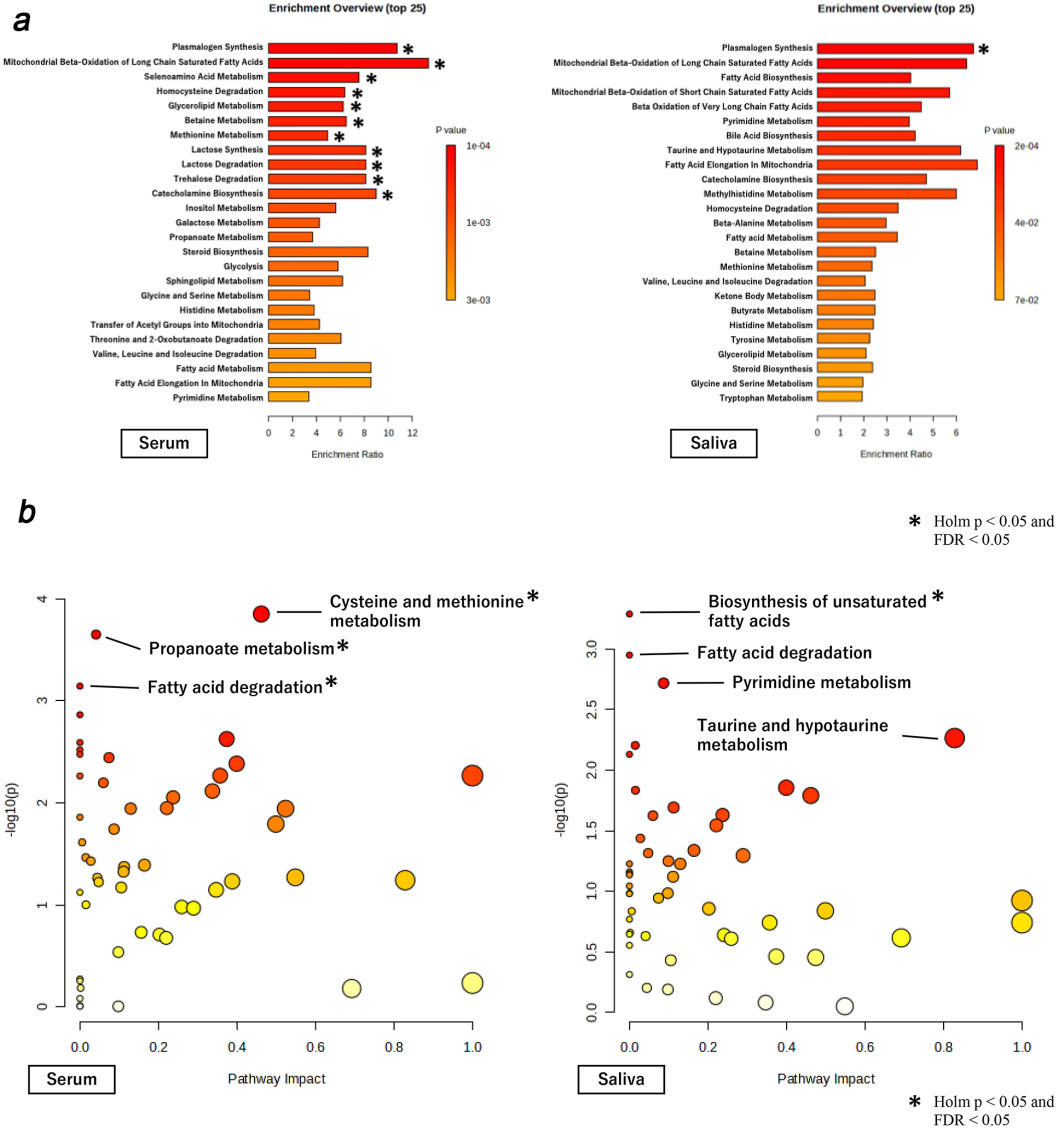
**a** Enrichment analysis of correlation between physical fatigue and serum and saliva metabolome. Enrichment analysis showing significant metabolic pathways correlated with physical fatigue in both serum and saliva samples. Pathways were identified through metabolomic profiling. An asterisk (*) indicates significance at Holm *p* < 0.05 and FDR < 0.05. **b** Pathway analysis of correlation between physical fatigue and serum and saliva metabolome. Pathway analysis illustrating the metabolic pathways associated with physical fatigue, including cysteine and methionine metabolism, propanoate metabolism, and fatty acid degradation. An asterisk (*) indicates significance at Holm *p* < 0.05 and FDR < 0.05. *Abbreviations* FDR, False Discovery Rate

In the analysis of physical fatigue (Fig. 1a and b), several significant pathways were identified in the blood samples, including plasmalogen synthesis (Holm *p* = 0.009, FDR = 0.003), mitochondrial beta-oxidation of long-chain saturated fatty acids (Holm *p* = 0.009, FDR = 0.003), and selenoamino acid metabolism (Holm *p* = 0.010, FDR = 0.003). In the saliva samples, enrichment analysis identified significant associations between plasmalogen synthesis (Holm *p* = 0.021, FDR = 0.021) and the biosynthesis of unsaturated fatty acids (Holm *p* = 0.027, FDR = 0.027) (Fig. 1a and b).

**Fig. 2:**
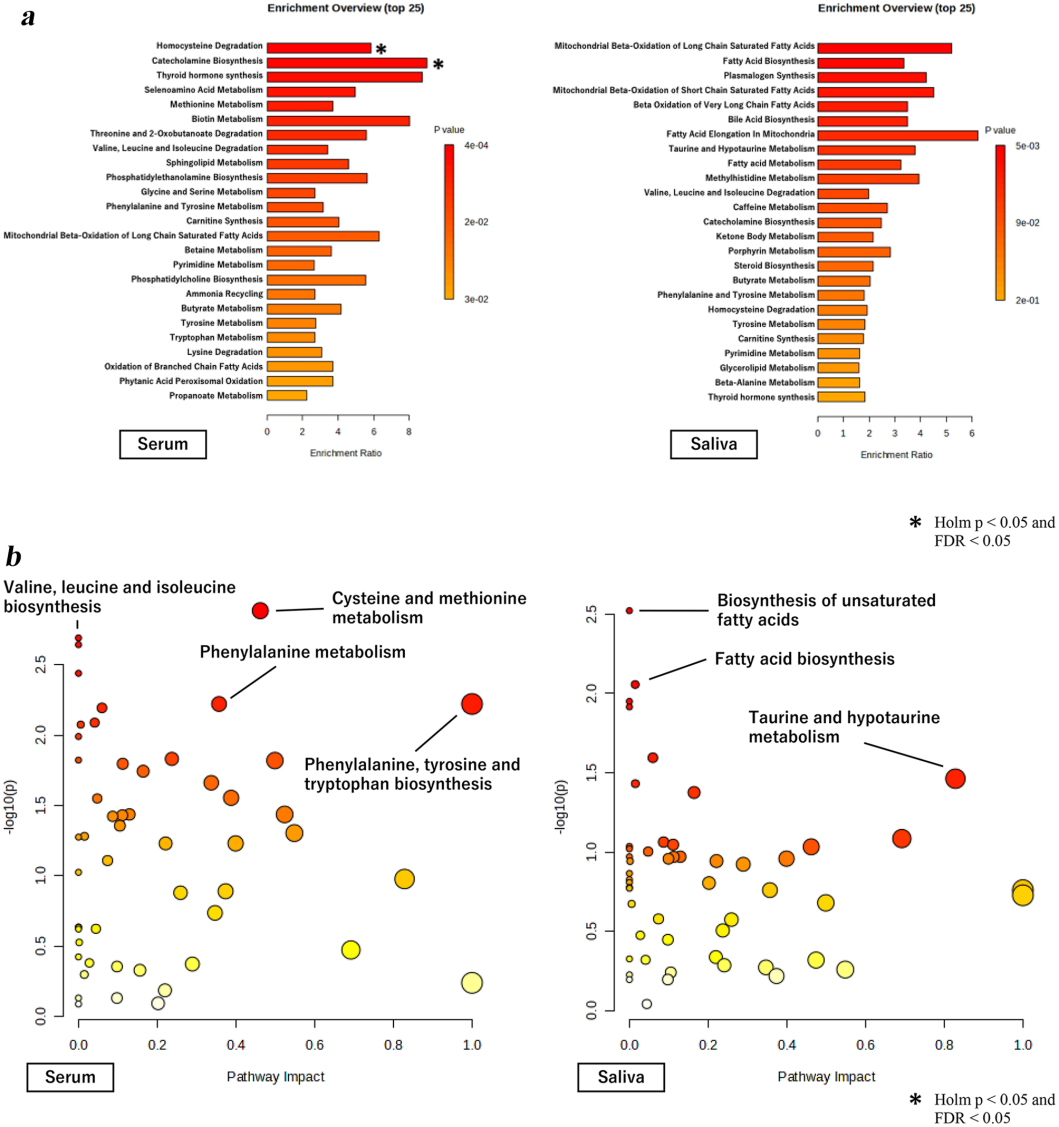
**a** Enrichment analysis of correlation between mental fatigue and serum and saliva metabolome. Enrichment analysis for pathways significantly associated with mental fatigue based on serum and saliva metabolomic profiles. An asterisk (*) indicates significance at Holm *p* < 0.05 and FDR < 0.05. **b** Pathway analysis of correlation between mental fatigue and serum and saliva metabolome. Pathway analysis for mental fatigue, highlighting key pathways such as cysteine and methionine metabolism, valine, leucine, and isoleucine biosynthesis. An asterisk (*) indicates significance at Holm *p* < 0.05 and FDR < 0.05

For mental fatigue, analysis of the blood sample revealed two significant pathways: homocysteine degradation (Holm *p* = 0.039, FDR = 0.022) and catecholamine biosynthesis (Holm *p* = 0.043, FDR = 0.022), as shown in Fig. 2a and b. No significant pathways for mental fatigue were identified in the saliva samples.

For general fatigue (Supplemental Figure S1a and b), a significant pathway was identified in the saliva samples: the biosynthesis of unsaturated fatty acids, which was significantly associated with general fatigue (Holm *p* = 0.044, FDR = 0.044). In the blood samples, no pathways were significantly associated with general fatigue.

Regarding reduced activity, neither the blood nor saliva samples showed significant pathways (Supplemental Figure S2a and b).

For reduced activity, neither the blood nor saliva samples showed significant pathways (Supplemental Figure S3a and b).

Finally, in the analysis of the total MFI score, as shown in Supplemental Figure S4a and b, blood samples showed a suggestive trend toward an association with homocysteine degradation (Holm *p* = 0.057, FDR = 0.032), although this did not reach statistical significance. No significant pathways were identified in the saliva samples for the total MFI score.

**Fig. 3:**
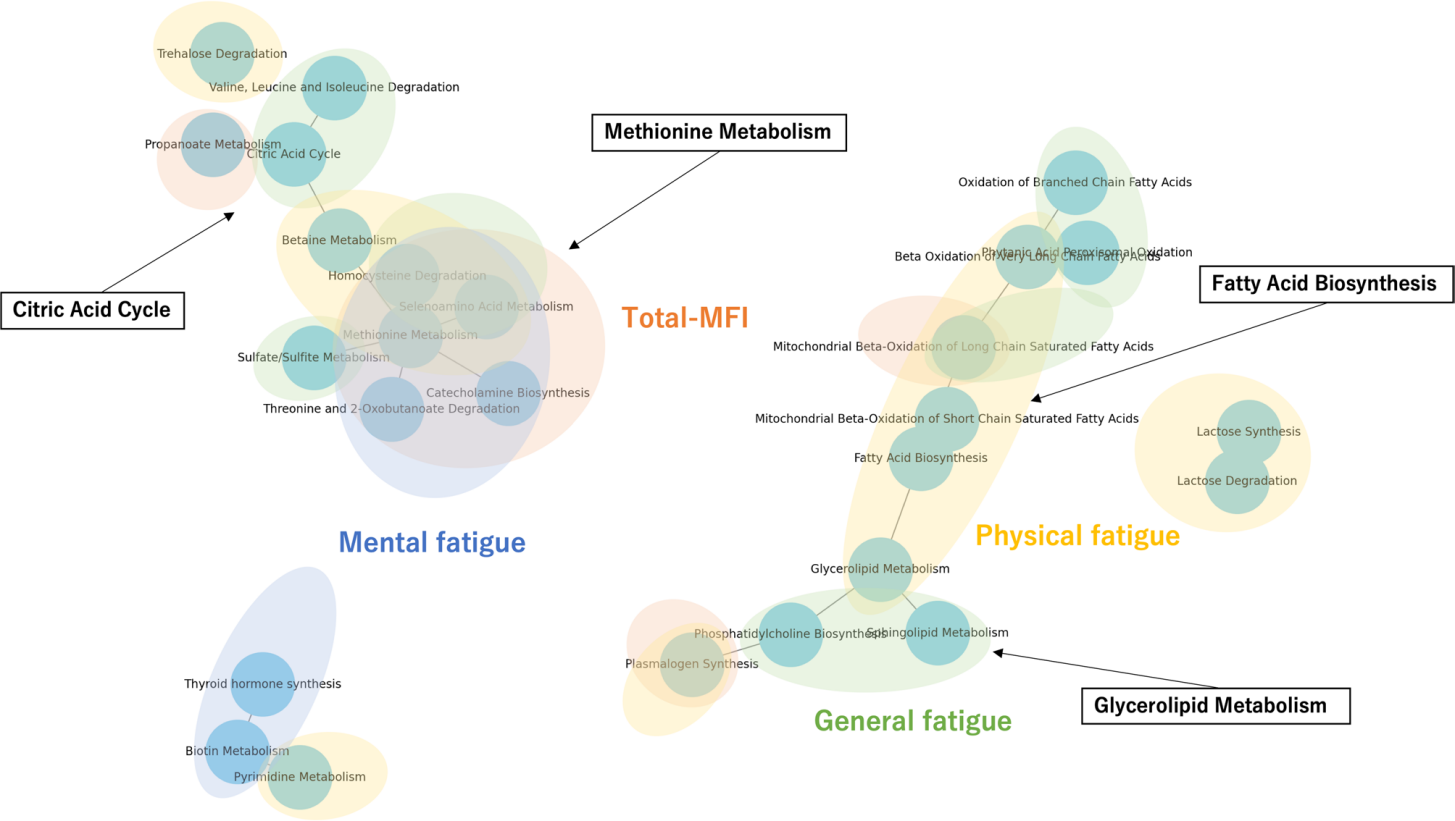
Pathway visualization of correlations between fatigue dimensions and metabolome in serum and saliva. Integrated visualization of metabolic pathways correlated with different dimensions of fatigue, based on serum and saliva samples

These findings, shown in Fig. 3, provide a comprehensive view of the metabolic pathways contributing to various dimensions of fatigue, including the total MFI score, physical fatigue, mental fatigue, and general fatigue.

### Microbiome and fatigue

Microbiome analysis identified several bacterial taxa associated with different fatigue dimensions. As shown in Table 3, a negative correlation was observed between *Firmicutes negaviticutes* and general fatigue (*r* = −0.379, *p* = 0.006). Additionally, *Patescibacteria saccharimonadia* was inversely correlated with both general (*r* = −0.306, *p* = 0.028) and physical (*r* = −0.304, *p* = 0.029) fatigue. Mental fatigue was significantly associated with *Actinobacteria* (*r* = −0.336, *p* = 0.015) and *Fusobacteria* (*r* = −0.285, *p* = 0.040). Several bacterial taxa showed weak-to-moderate correlations with fatigue scores, with correlation coefficients ranging from −0.285 to −0.379. Although these correlations are relatively small, they may provide preliminary insights into potential microbiome-fatigue associations, warranting further investigation in larger cohorts.

**Table 3:** Correlations between fatigue and saliva microbiome

	General fatigue *r* (*p*-value)	Physical fatigue *r* (*p*-value)	Mental fatigue *r* (*p*-value)	Reduced activity *r* (*p*-value)	Reduced motivation *r* (*p*-value)	Total MFI score *r* (*p*-value)
Actinobacteriota actinobacteria	−0.279 (0.045)	−0.296 (0.033)	−0.336 (0.015)		−0.276 (0.048)	−0.322 (0.020)
Fusobacteriota fusobacteriia			−0.285 (0.040)			
Firmicutes negativicutes	−0.379 (0.006)	−0.385 (0.005)			−0.341 (0.013)	−0.369 (0.007)
Patescibacteria saccharimonadia	−0.306 (0.028)	−0.304 (0.029)			−0.284 (0.041)	
Verrucomicrobiota verrucomicrobiae		0.343 (0.013)		0.429 (0.001)		0.315 (0.023)
Euryarchaeota methanobacteria		0.343 (0.013)		0.429 (0.001)		0.315 (0.023)

Pearson correlation coefficients (*r*) and corresponding *p*-values indicating associations between fatigue dimensions (general fatigue, physical fatigue, mental fatigue, reduced activity, reduced motivation, and total MFI score) and specific bacterial taxa in the salivary microbiome. *Abbreviations* MFI, Multidimensional Fatigue Inventory; SCFA, Short-Chain Fatty Acid

We also examined the associations between fatigue dimensions and both alpha (Shannon and Simpson indices) and beta (Dim1 and Dim2 from the MDS analysis) diversities. No significant correlations were observed between fatigue and diversity indices (Supplemental Table S1).

### Genetic associations

Genetic analysis identified several single nucleotide polymorphisims (SNPs) that were significantly associated with various dimensions of fatigue, shedding light on the potential genetic pathways involved in energy regulation, neurotransmitter signaling, and metabolic processes. Table 4 lists the top 10 SNPs for each fatigue dimension along with their related genes and provides a detailed overview of all SNPs significantly associated with fatigue dimensions.

**Table 4:** Top 10 significant correlations between fatigue and SNP

General fatigue SNP *r* (*p*-value)	Physical fatigue SNP *r* (*p*-value)	Mental fatigue SNP *r* (*p*-value)	Reduced activity SNP *r* (*p*-value)	Reduced motivation SNP *r* (*p*-value)	Total MFI score SNP *r* (*p*-value)
rs891762 −0.539 (0.001)	rs71249850 0.470 (0.007)		rs9556404 −0.552 (0.001)	rs9556404 −0.448 (0.010)	rs9556404 −0.523 (0.002)
rs28654511 0.508 (0.003)	rs9556404 −0.449 (0.010)		rs17053446 0.517 (0.002)	rs78847973 −0.395 (0.025)	rs28654511 0.473 (0.006)
rs215539 0.501 (0.004)	rs28654511 0.442 (0.011)		rs3896184 0.509 (0.003)	rs3816836 0.366 (0.039)	rs3896184 0.468 (0.007)
rs1332664 0.484 (0.005)	rs12292548 0.425 (0.015)		rs2074621 0.498 (0.004)	rs71249850 0.366 (0.040)	rs71249850 0.452 (0.009)
rs8069305 0.475 (0.006)	rs7455704 0.417 (0.018)		rs2274951 −0.482 (0.005)	rs2074621 0.355 (0.046)	rs2074621 0.442 (0.011)
rs3896184 0.470 (0.007)	rs6539183 −0.394 (0.026)		rs2360170 −0.473 (0.006)	rs9808231 0.353 (0.047)	rs891762 −0.429 (0.014)
rs9556404 −0.465 (0.007)	rs891762 −0.387 (0.029)		rs2092940195 −0.461 0.008		rs472391 0.426 (0.015)
rs13409950 −0.454 (0.009)	rs8069305 0.383 (0.031)		rs472391 0.459 (0.008)		rs2092940195 −0.426 (0.015)
rs2074621 0.438 (0.012)	rs2092940195 −0.378 (0.033)		rs12292548 0.454 (0.009)		rs2274951 −0.418 (0.017)
rs1840570 −0.420 (0.017)	rs3896184 0.367 (0.039)		rs2172250 −0.447 (0.010)		rs8069305 0.405 (0.021)

Top ten SNPs significantly correlated with each fatigue dimension (general fatigue, physical fatigue, mental fatigue, reduced activity, reduced motivation, and total MFI score). Pearson correlation coefficients (*r*) and *p*-values are shown for each SNP associated with fatigue scores. *Abbreviations* MFI, Multidimensional Fatigue Inventory; SNP, Single Nucleotide Polymorphism

For general fatigue, significant associations were observed between several SNPs; rs891762 (*r* = − 0.539, *p* = 0.001) in the *PLXNA1* gene, which is involved in plexin-mediated signaling pathways, [21] and rs28654511 (*r* = 0.508, *p* = 0.003) in *HSD17B11*, a gene involved in steroid hormone metabolism [22], were among the top findings. Additionally, rs215539 (*r* = 0.501, *p* = 0.004) in *CPXM1*, which is associated with tumor progression and immune cell infiltration [23], and rs1332664 (*r* = 0.484, *p* = 0.005) in *CFHR5*, a gene related to complement regulation [24], were also significantly associated with general fatigue. Additionally, rs8069305 (*r* = 0.475, *p* = 0.006) and rs3896184 (*r* = 0.470, *p* = 0.007) in *SVIL*, which are related to cytokinesis and cytoskeletal regulation, respectively [25]; rs9556404 (*r* = − 0.465, *p* = 0.007) in *GPR180*, associated with associated with thermogenic regulation and transformaing growth factor beta (TGF-β) signaling [26], rs2074621 (*r* = 0.438, *p* = 0.012) in *NOTCH3*, involved in vascular development [27], and rs1840570 (*r* = − 0.420, *p* = 0.017) in *GUCY1A2*, involved in cGMP synthesis [28], were identified as significant contributors to general fatigue.

The most significant SNPs for physical fatigue were rs71249850 (*r* = 0.470, *p* = 0.007) and rs9556404 in *GPR180*. Other notable associations included rs28654511 and rs12292548 (*r* = 0.425, *p* = 0.015) in *CNTN5*, which are involved in neuronal cell adhesion [29], and rs7455704 (*r* = 0.417, *p* = 0.018) and rs6539183 (*r* = − 0.394, *p* = 0.026) in *ALDH1L2*, which are associated with mitochondrial one-carbon metabolism and NADP(+)-dependent conversion of 10-formyltetrahydrofolate [30]. Additionally, rs891762 in *PLXNA1*, rs2092940195 (*r* = −0.378, *p* = 0.033) in *PKD1*, which is associated with muscle performance enhancement and *MEF2* gene activation in skeletal muscle [31], and rs3896184 in *SVIL* were significantly associated with physical fatigue. For mental fatigue, no SNPs were found to be significantly associated.

For reduced activity, significant associations were found for rs9556404 in *GPR180*, rs17053446 (*r* = 0.517, *p* = 0.002) in *DOCK5*, which is involved in cytoskeletal dynamics [32], and rs3896184 in *SVIL*. Additionally, rs2074621 in *NOTCH3*, rs2274951 (*r* = −0.482, *p* = 0.005) in *PELATON*, which is associated with spermatogenesis and mRNA QC [33], and rs2360170 (*r* = −0.473, *p* = 0.006) were significantly associated.

For reduced motivation, rs9556404 (*r* = −0.523, *p* = 0.002) in *GPR180*, rs28654511 (*r* = 0.473, *p* = 0.006) in *HSD17B11*, and rs3896184 (*r* = 0.468, *p* = 0.007) in *SVIL* were associated. Other SNPs, such as rs71249850 (*r* = 0.452, *p* = 0.009) and rs2074621 (*r* = 0.442, *p* = 0.011) in *NOTCH3*, as well as rs891762 (*r* = −0.429, *p* = 0.014) in *PLXNA1*, were significant.

### Prediction models for physical and mental fatigue

**Fig. 4:**
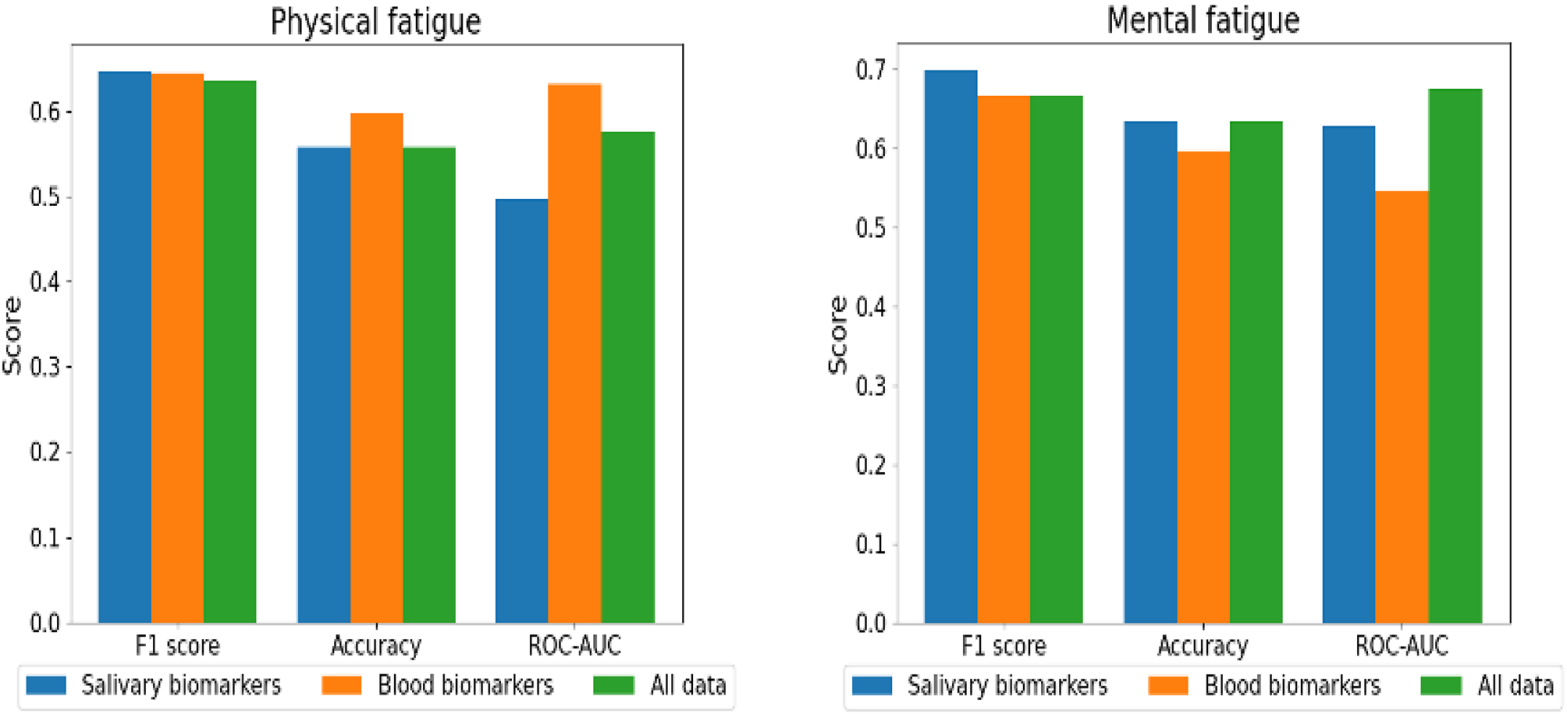
Performance metrics of machine learning models using blood and salivary biomarkers for predicting physical and mental fatigue. Prediction models were developed using LightGBM, a gradient boosting decision tree algorithm, to classify physical and mental fatigue in a cohort of 52 patients with non-communicable diseases (NCDs). Models were constructed using three sets of explanatory variables: (1) salivary biomarkers, (2) blood biomarkers, and (3) a combined dataset incorporating both biomarker types, along with age, NCD category, and microbiome data. Fatigue classifications were based on the Japanese version of the Multidimensional Fatigue Inventory (MFI), with thresholds set at mean scores of 10.7 for physical fatigue and 10.6 for mental fatigue. Model performance was evaluated using F1 scores, accuracy, and receiver operating characteristic area under the curve (ROC-AUC), with leave-one-out cross-validation applied. Results indicated moderate performance across models, with blood biomarker models showing higher predictive capability for physical fatigue (F1 scores: 0.63–0.65, accuracy: 0.56–0.60, ROC-AUC: 0.50–0.63), and salivary biomarker models achieving relatively higher performance for mental fatigue (F1 scores: 0.67–0.70, accuracy: 0.60–0.63, ROC-AUC: 0.55–0.67)

As shown in Fig. 4, the prediction models demonstrated moderate performances for both physical and mental fatigue. For physical fatigue, model performance included F1 scores between 0.63 and 0.65, accuracy between 0.56 and 0.60, and ROC-AUC values between 0.50 and 0.63. Notably, the model using blood biomarkers as explanatory variables achieved a relatively higher performance than the other models. We calculated SHapley Additive exPlanation (SHAP) values [34] for the prediction model and observed that biomarkers, such as 3-hydroxyisovaleric acid and uridine, exhibited high absolute SHAP values (Supplemental Figure S5a). For mental fatigue, model performance yielded F1 scores between 0.67 and 0.70, accuracy between 0.60 and 0.63, and ROC-AUC values between 0.55 and 0.67, with the model utilizing salivary biomarkers showing relatively higher performance. We calculated SHAP values for the prediction model and observed that biomarkers, such as orotic acid and lactitol, exhibited high absolute SHAP values (Supplemental Figure S5b). Additionally, we explored additional machine learning algorithms other than LightGBM for predicting physical and mental fatigue. Specifically, we examined logistic regression and RUSBoostClassifier [35], the latter of which is designed to handle imbalanced datasets. While LightGBM still demonstrated the best performance in predicting mental fatigue, these methods showed comparable prediction performance to LightGBM (shown in Supplemental Table S2). These results suggest that both salivary and blood biomarkers may have potential to classify and predict fatigue, although further validation is required to improve their predictive performance.

## Discussion

This study explored the biological mechanisms underlying fatigue in patients with NCDs using a multi-omics approach. Our findings indicate that distinct metabolic pathways, salivary microbiota, and genetic factors may contribute to different dimensions of fatigue, including general, physical, and mental fatigue. General fatigue is associated with unsaturated fatty acid biosynthesis, indicating its role in lipid metabolism. Physical fatigue was associated with plasmalogen synthesis, mitochondrial beta-oxidation of long-chain fatty acids, and selenoamino acid metabolism, suggesting a potential contribution of impaired energy production. Mental fatigue is associated with homocysteine degradation and catecholamine biosynthesis, which may influence cognitive fatigue. Microbiome analysis indicated that lower levels of *Firmicutes negativicutes* were associated with general fatigue, whereas *Patescibacteria saccharimonadia* showed an inverse correlation with both general and physical fatigue. Genetic analysis identified SNPs in genes, such as *GPR180, NOTCH3, SVIL, HSD17B11*, and *PLXNA1* that may be involved in pathways related to energy metabolism, neurotransmitter signaling, and cellular function. Our findings suggest that integrating multi-omics analyses with diverse biological samples may provide valuable insights into the mechanisms underlying fatigue. Although this study offers preliminary insights, further validation in larger and more diverse cohorts is necessary to confirm these associations and explore potential clinical applications.

### Energy metabolism and fatigue: the key role of lipid and Amino acid pathways

Disruptions in lipid metabolism, particularly in unsaturated fatty acid biosynthesis, are associated with both general and physical fatigue, indicating their potential role in energy balance. Additionally, fatty acid oxidation and plasmalogen synthesis were more specifically linked to physical fatigue, consistent with previous findings regarding the role of lipid metabolism in energy regulation [36, 37, 38]. These disruptions have been reported in patients with CFS, diabetes, cardiovascular disease, and neurodegenerative disorders [36, 37, 38]. Although our findings support the role of lipid metabolism, the contribution of plasmalogen synthesis remains underexplored. Further studies are needed to validate these findings and understand their mechanisms of action.

Mental fatigue was associated with homocysteine degradation and catecholamine biosynthesis, which suggests a link between oxidative stress and neurotransmitter regulation. Previous studies [38, 39] have reported that elevated homocysteine levels are related to cognitive decline and fatigue, indicating that disruptions in these pathways could contribute to mental fatigue by affecting cognitive function and stress responses.

### Dysbiosis of salivary microbiome is associated with fatigue

Our microbiome analysis revealed a depletion of *Firmicutes negaviticutes* and *Patescibacteria saccharimonadia*, suggesting that microbial dysbiosis may be linked to physical and general fatigue. These taxa influence immune function and systemic inflammation [40], both of which have been implicated in fatigue [36, 37]. Our findings suggest a possible role of the salivary microbiome in systemic inflammation and fatigue in patients with NCDs. Although previous studies have focused on the gut microbiota in fatigue-related conditions, such as CFS and inflammatory bowel disease [41, 42], our study provides preliminary insights into the role of the oral microbiome in NCD-related fatigue. Future studies should investigate the diagnostic potential of the salivary microbiota as a marker of systemic inflammation. Although alpha and beta diversities did not correlate with fatigue, specific microbial taxa, such as *Firmicutes negativicutes* and *Patescibacteria saccharimonadia*, showed significant associations, suggesting that microbial composition, rather than overall diversity, may be a key factor in fatigue. Previous studies have indicated that microbial stability is reflected by alpha and beta diversities; however, the functional effects of microbial communities are often determined by specific taxa with unique metabolic or immunological roles [7]. Based on this finding, specific microbiota groups, such as *Firmicutes* and *Patescibacteria*, rather than general microbial diversity, may play a role in the relationship between dysbiosis and fatigue.

### Genetic contributions to fatigue dimensions

Genetic analysis identified SNPs that were potentially associated with different dimensions of fatigue, including cognitive, physical, and motivational aspects. However, *GPR180, HSD17B11, PLXNA1, NOTCH3,* and *SVIL* were observed across multiple fatigue dimensions, warranting further investigation. The *GPR180* gene, involved in thermogenic regulation and TGF-β signaling [26], was associated with both general and physical fatigue. This gene regulates the energy balance, and its dysregulation may contribute to fatigue [36, 37]. Similarly, *HSD17B11* and *PLXNA1* have been linked to steroid hormone metabolism [22] and plexin-mediated signaling [21], both of which may affect systemic energy regulation and resilience to fatigue [36, 37]. The *NOTCH3* gene, known for its role in vascular development [27], is linked to multiple fatigue factors, possibly because of its function in maintaining oxygen and nutrient supplies to tissues, which is critical for mental and physical energy levels [43]. The *SVIL* gene, which plays a role in the integrity of cytokinesis and cytoskeletal [25], may be related to physical and cognitive fatigue by supporting cellular structure under stress [44]. Our findings suggest that genetic factors may interact with metabolic and immune pathways in fatigue development. Further studies are needed to elucidate these mechanisms and determine whether they vary across different chronic conditions.

### Integration of findings: a multifactorial mechanism in fatigue

Fatigue in patients with NCDs may result from interactions between inflammation, metabolic dysregulation, and cellular integrity. Microbial imbalances may trigger inflammatory responses, potentially leading to mitochondrial dysfunction and impaired energy balance [37, 45] which may play a role in the development of fatigue. Genetic predispositions, particularly those affecting metabolic pathways, neurotransmitter signaling, and vascular health, can further affect energy production and cellular resilience [46]. Additionally, imbalances in lipid and amino acid metabolism may limit energy availability, thereby reinforcing multilevel fatigue [47, 48]. Our findings suggest that microbiome composition, metabolic pathways, and genetic variability may contribute to fatigue in patients with NCDs. These findings suggest possible therapeutic directions; however, it is important to note that the observed associations are correlational. Causal inferences cannot be drawn from this cross-sectional design, and further longitudinal and interventional research is necessary to investigate whether these biological markers contribute to fatigue development or progression.

### Comparison with fatigue in other diseases

The biological mechanisms underlying fatigue in patients characterized by metabolic dysfunction, microbial dysbiosis, and genetic predispositions, may share similarities with other conditions, such as CFS, cancer-related fatigue (CRF), and autoimmune diseases such as systemic lupus erythematosus (SLE). However, direct comparisons remain speculative and require further investigations.

Disruptions in the citric acid cycle and lipid metabolism have been reported in CFS, suggesting that impaired energy production is a common feature of fatigue-related disorders [36, 37]. Mitochondrial dysfunction, leading to reduced ATP availability, has also been implicated in CFS and NCD-related fatigue [36]. However, our findings suggest that unsaturated fatty acid biosynthesis may play a role in NCD-related fatigue, an aspect that has been less explored in CFS where studies have primarily focused on oxidative metabolic pathways, including fatty acid oxidation.

Aberrant metabolism is well documented in CRF, particularly disruptions in glycolysis and oxidative phosphorylation, as tumor cells exhibit increased energy demands [48]. Although both CRF- and NCD-related fatigue involve metabolic dysfunction, CRF is more closely associated with glycolysis [49]. Our findings emphasize the role of lipid metabolism in NCD-related fatigue. These differences suggest that although metabolic dysregulation is a shared feature, the specific pathways involved may differ, depending on the underlying disease.

In autoimmune diseases, such as SLE, immune dysregulation, and chronic inflammation, are key contributors to fatigue [50, 51]. Although we did not observe significant associations with individual inflammatory cytokines, such as interleukin-6 or tumour necrosis factor alpha in our study, microbial dysbiosis may still contribute to fatigue through indirect inflammatory mechanisms. Additionally, genetic predispositions affecting neurotransmitter signaling and vascular function may contribute to fatigue in patients with NCDs, possibly through distinct mechanisms compared with autoimmune diseases. Further studies are needed to determine whether inflammation-related pathways differ between these conditions.

### Prospects for blood- and salivary-based fatigue prediction

Our findings suggest that blood and salivary biomarkers may have the potential to predict different dimensions of fatigue in patients with NCDs. Notably, blood-based models showed a higher predictive performance for physical fatigue, whereas salivary models appeared to be more indicative of mental fatigue. This suggests that biological sample selection can be optimized, depending on whether the focus is on physical or mental fatigue. However, these findings require further validation using larger cohorts. The predictive performance of our models was moderate (F1 scores of 0.65 for physical fatigue and 0.70 for mental fatigue), indicating that although blood and saliva biomarkers may be useful in fatigue assessment, additional refinement is necessary before clinical application. Salivary biomarkers appear to reflect psychological rather than physical fatigue status, whereas blood-based biomarkers are more closely associated with physiological processes. The development of the prediction models presented in this study is an initial exploration. We plan to conduct further investigations to improve their performance in future large-scale study. A more systematic selection of biological samples may improve fatigue prediction models. Future studies should explore alternative machine learning approaches, such as deep learning [52, 53], which may capture more complex relationships between biomarker features, and statistical approaches better suited for small samples, such as Bayesian methods, regularization techniques, and feature selection. Additionally, incorporating clinical and behavioral factors, including sleep quality, stress levels, and physical activity, could improve predictive accuracy and clinical applicability. Saliva may offer a practical, non-invasive option for longitudinal fatigue monitoring, providing a less burdensome alternative for patients. The combination of salivary and blood biomarkers may contribute to a more comprehensive fatigue assessment model. However, further studies are required to determine the feasibility of integrating these biomarkers in clinical practice.

### Limitations

This study provides preliminary insights into the biological correlates of fatigue in patients with NCDs; however, several limitations must be acknowledged. First, the small sample size is a major limitation of this study. No formal power analysis was performed; therefore, the results should be considered exploratory and interpreted with caution. Multiple comparison corrections were applied to reduce the likelihood of false positives. The LightGBM algorithm, which includes built-in feature selection and regularization, was selected to handle high-dimensional data in a small sample context. Nonetheless, larger and more powered studies will be essential to validate these findings and improve generalizability. Second, although our findings suggest potential relationships between metabolic pathways, salivary microbiota, and genetic factors, causal relationships cannot be established. Further studies, including experimental models, are required to validate these potential causal pathways. As this study was conducted in a Japanese population, the findings may not be generalizable to other ethnic groups. Genetic and environmental differences may influence fatigue mechanisms, necessitating further studies in diverse cohorts. Additionally, most participants were older adults, indicating that the observed metabolic and microbial responses may be age-dependent. Future studies should explore whether these findings are applicable to younger populations. Furthermore, when compared to healthy individuals reported in the previous study [18], the fatigue levels observed in our study participants with NCDs were not markedly higher. This suggests that our cohort did not represent a population experiencing severe fatigue. This limitation should be considered when interpreting the findings. However, despite this, the use of the MFI remains valuable, as it allows for the classification of fatigue into five distinct domains. This detailed categorization provides insights into the specific characteristics of fatigue in individuals with NCDs, which may not be captured through a single fatigue score alone. Finally, the cross-sectional nature of this study precludes an assessment of temporal dynamics. Longitudinal studies are essential to clarify how biological perturbations contribute to fatigue progression. Despite these limitations, this study offers a foundation for future studies on fatigue mechanisms, thereby highlighting potential biomarkers that warrant further investigation in larger and more diverse populations.

## Conclusion

This exploratory study suggests that fatigue in patients with NCDs may involve disruptions in lipid metabolism, neurotransmitter pathways, microbial composition, and genetic variations. Our multi-omics machine learning approach provides preliminary insights that require further validation. Notably, blood-based biomarkers showed better predictive potential for physical fatigue, whereas salivary-based models were more indicative of mental fatigue. These findings may contribute to the development of fatigue assessment models applicable to other chronic diseases.

This exploratory study provides preliminary, correlational insights into potential biological contributors to fatigue in NCD patients. Further validation, including longitudinal designs and interventional trials, will be essential to determine causality and clinical utility.

## Electronic supplementary material

Below is the link to the electronic supplementary material.

Supplementary Material 1

Supplementary Material 2

Supplementary Material 3

Supplementary Material 4

## Data Availability

The datasets used and/or analysed during the current study are available from the corresponding author on reasonable request.
