# Supplementary material for: Visualizing fatigue mechanisms in non-communicable diseases: an integrative approach with multi-omics and machine learning
Source: BMC Med Inform Decis Mak. 2025 Jun 3;25:204. doi: 10.1186/s12911-025-03034-3 (PMC12135302; doi:10.1186/s12911-025-03034-3)
Supplement: Supplementary file 1 — Supplementary Material 1 [file 12911_2025_3034_MOESM1_ESM.docx]

**Figure legends**

**Figure 1a. Enrichment analysis of correlation between physical fatigue and serum and saliva metabolome.**
Enrichment analysis showing significant metabolic pathways correlated with physical fatigue in both serum and saliva samples. Pathways were identified through metabolomic profiling. An asterisk (*) indicates significance at Holm p < 0.05 and FDR < 0.05. Abbreviations: FDR, False Discovery Rate.

**Figure 1b. Pathway analysis of correlation between physical fatigue and serum and saliva metabolome.**
Pathway analysis illustrating the metabolic pathways associated with physical fatigue, including cysteine and methionine metabolism, propanoate metabolism, and fatty acid degradation. An asterisk (*) indicates significance at Holm p < 0.05 and FDR < 0.05.

**Figure 2a. Enrichment analysis of correlation between mental fatigue and serum and saliva metabolome.**
Enrichment analysis for pathways significantly associated with mental fatigue based on serum and saliva metabolomic profiles. An asterisk (*) indicates significance at Holm p < 0.05 and FDR < 0.05.

**Figure 2b. Pathway analysis of correlation between mental fatigue and serum and saliva metabolome.**
Pathway analysis for mental fatigue, highlighting key pathways such as cysteine and methionine metabolism, valine, leucine, and isoleucine biosynthesis. An asterisk (*) indicates significance at Holm p < 0.05 and FDR < 0.05.

**Figure 3. Pathway visualization of correlations between fatigue dimensions and metabolome in serum and saliva.**
Integrated visualization of metabolic pathways correlated with different dimensions of fatigue, based on serum and saliva samples.

**Figure 4.** **Performance Metrics of Machine Learning Models Using Blood and Salivary Biomarkers for Predicting Physical and Mental Fatigue.**

Prediction models were developed using LightGBM, a gradient boosting decision tree algorithm, to classify physical and mental fatigue in a cohort of 52 patients with non-communicable diseases (NCDs). Models were constructed using three sets of explanatory variables: (1) salivary biomarkers, (2) blood biomarkers, and (3) a combined dataset incorporating both biomarker types, along with age, NCD category, and microbiome data. Fatigue classifications were based on the Japanese version of the Multidimensional Fatigue Inventory (MFI), with thresholds set at mean scores of 10.7 for physical fatigue and 10.6 for mental fatigue. Model performance was evaluated using F1 scores, accuracy, and receiver operating characteristic area under the curve (ROC-AUC), with leave-one-out cross-validation applied. Results indicated moderate performance across models, with blood biomarker models showing higher predictive capability for physical fatigue (F1 scores: 0.63–0.65, accuracy: 0.56–0.60, ROC-AUC: 0.50–0.63), and salivary biomarker models achieving relatively higher performance for mental fatigue (F1 scores: 0.67–0.70, accuracy: 0.60–0.63, ROC-AUC: 0.55–0.67).
