# Supplementary material for: Visualizing fatigue mechanisms in non-communicable diseases: an integrative approach with multi-omics and machine learning
Source: BMC Med Inform Decis Mak. 2025 Jun 3;25:204. doi: 10.1186/s12911-025-03034-3 (PMC12135302; doi:10.1186/s12911-025-03034-3)
Supplement: Supplementary file 2 — Supplementary Material 2 [file 12911_2025_3034_MOESM2_ESM.docx]

**Supplemental Figure Legends**

**Supplemental Figure S1a. Enrichment analysis of correlation between general fatigue and serum and saliva metabolome.**
Enrichment analysis showing pathways associated with general fatigue across serum and saliva, identifying significant enrichment patterns. An asterisk (*) indicates significance at Holm p < 0.05 and FDR < 0.05.

**Supplemental Figure S1b. Pathway analysis of correlation between general fatigue and serum and saliva metabolome.**
Pathway analysis of general fatigue, featuring pathways like propanoate metabolism and pentose and glucuronate interconversions. An asterisk (*) indicates significance at Holm p < 0.05 and FDR < 0.05.

**Supplemental Figure S2a. Enrichment analysis of correlation between reduced activity and serum and saliva metabolome.**
Enrichment analysis for pathways correlated with reduced activity in serum and saliva, highlighting key metabolic associations. An asterisk (*) indicates significance at Holm p < 0.05 and FDR < 0.05.

**Supplemental Figure S2b. Pathway analysis of correlation between reduced activity and serum and saliva metabolome.**
Pathway analysis detailing metabolic pathways associated with reduced activity, including fatty acid biosynthesis and amino sugar metabolism. An asterisk (*) indicates significance at Holm p < 0.05 and FDR < 0.05.

**Supplemental Figure S3a. Enrichment analysis of correlation between reduced motivation and serum and saliva metabolome.**
Enrichment analysis identifying pathways associated with reduced motivation in serum and saliva samples. An asterisk (*) indicates significance at Holm p < 0.05 and FDR < 0.05.

**Supplemental Figure S3b. Pathway analysis of correlation between reduced motivation and serum and saliva metabolome.**
Pathway analysis revealing pathways correlated with reduced motivation, such as taurine and hypotaurine metabolism. An asterisk (*) indicates significance at Holm p < 0.05 and FDR < 0.05.

**Supplemental Figure S4a. Enrichment analysis of correlation between total MFI score and serum and saliva metabolome.**
Enrichment analysis showing metabolic pathways associated with the total MFI score, reflecting comprehensive fatigue assessment. An asterisk (*) indicates significance at Holm p < 0.05 and FDR < 0.05.

**Supplemental Figure S4b. Pathway analysis of correlation between total MFI score and serum and saliva metabolome.**
Pathway analysis illustrating pathways correlated with total fatigue scores, including cysteine and methionine metabolism, and taurine and hypotaurine metabolism. An asterisk (*) indicates significance at Holm p < 0.05 and FDR < 0.05.

**Supplemental Figure S5a. Bar plot showing the top 10 average of absolute SHAP values for the prediction model of physical fatigue using blood biomarkers.**

This figure presents the top blood metabolites contributing to physical fatigue based on SHAP (SHapley Additive exPlanations) values derived from a LightGBM model. The x-axis represents the mean absolute SHAP values, indicating the impact of each metabolite on the model’s prediction. Higher SHAP values suggest stronger contributions to physical fatigue. The most influential metabolites include 3-Hydroxyisovaleric acid, Uridine, Phosphoric acid, and Decanoic acid.

**Supplemental Figure S5b. Bar plot showing the top 10 average of absolute SHAP values for the prediction model of mental fatigue using saliva biomarkers.**

This figure illustrates the top salivary metabolites associated with mental fatigue as determined by SHAP values from a LightGBM model. The x-axis denotes the mean absolute SHAP values, representing the extent of each metabolite’s influence on mental fatigue prediction. Orotic acid and Lactitol show the highest contributions, followed by Palmitic acid, 3-Hydroxyisobutyric acid, and Isocitric acid.
