# Supplementary material for: Visualizing fatigue mechanisms in non-communicable diseases: an integrative approach with multi-omics and machine learning
Source: BMC Med Inform Decis Mak. 2025 Jun 3;25:204. doi: 10.1186/s12911-025-03034-3 (PMC12135302; doi:10.1186/s12911-025-03034-3)
Supplement: Supplementary file 3 — Supplementary Material 3 [file 12911_2025_3034_MOESM3_ESM.pptx]

## Slide 1
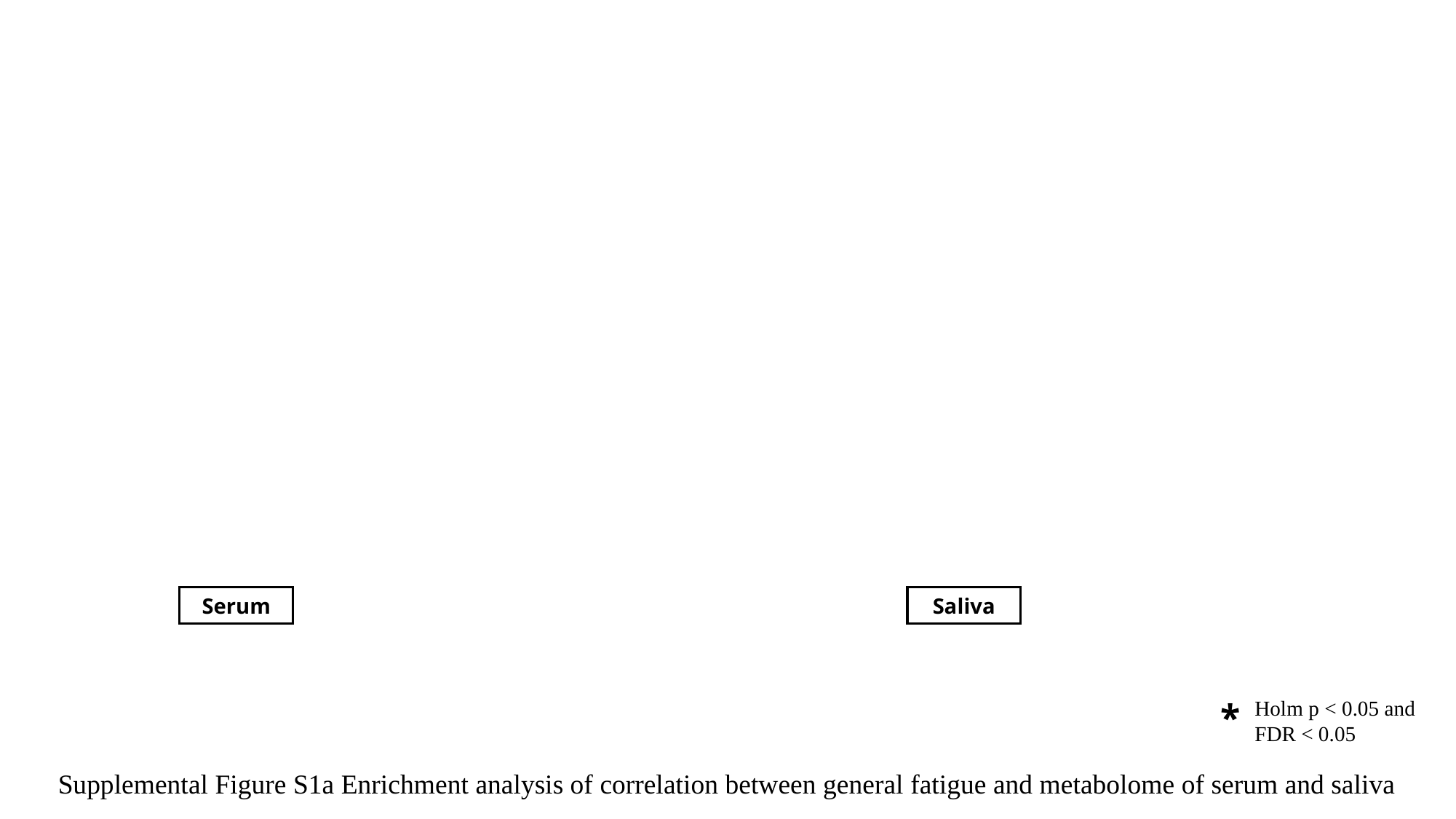

Serum
Saliva
*
Holm p < 0.05 and FDR < 0.05
Supplemental Figure S1a Enrichment analysis of correlation between general fatigue and metabolome of serum and saliva

## Slide 2
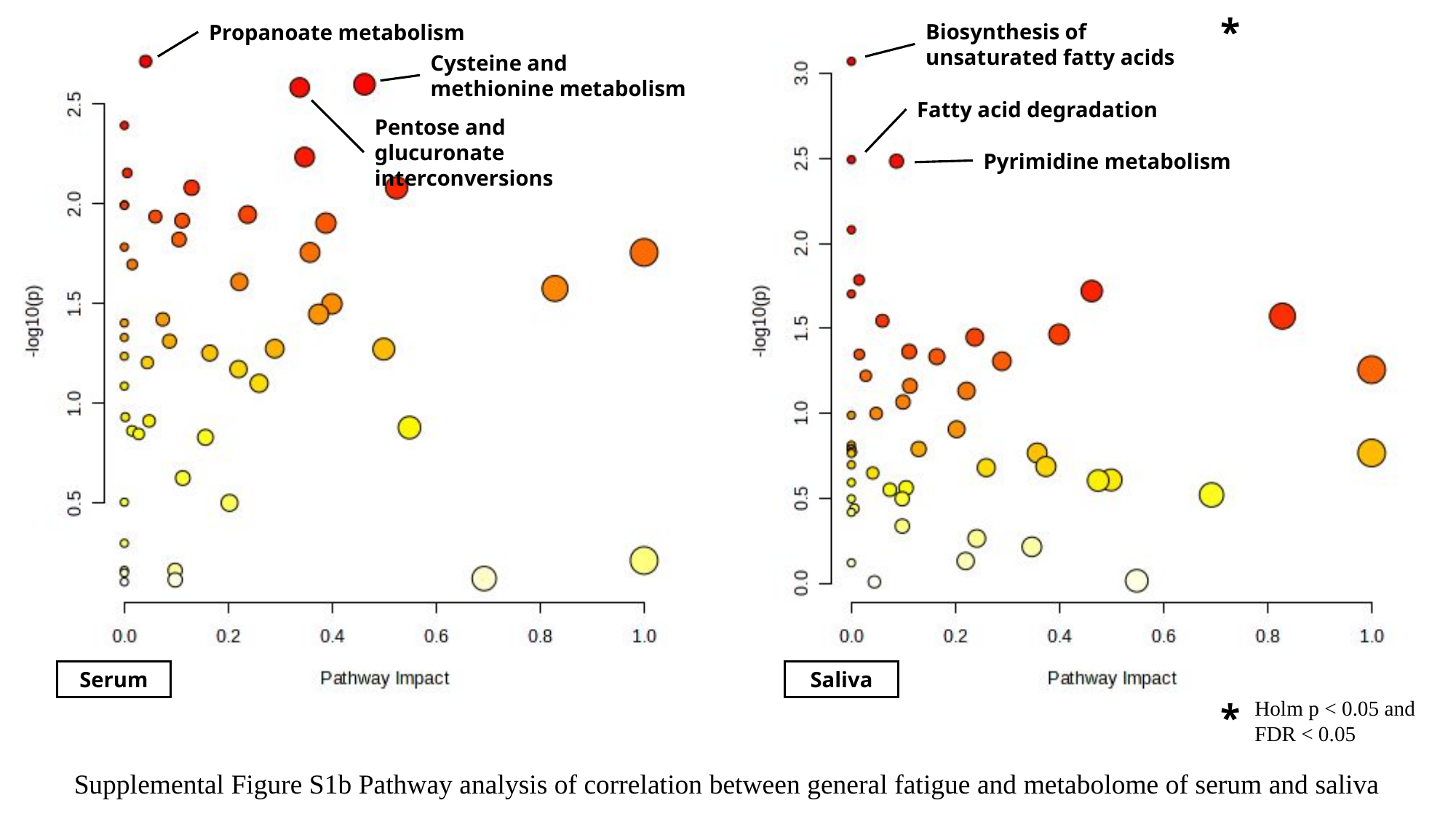

*
Biosynthesis of unsaturated fatty acids
Propanoate metabolism
Cysteine and methionine metabolism
Fatty acid degradation
Pentose and glucuronate interconversions
Pyrimidine metabolism
Serum
Saliva
*
Holm p < 0.05 and FDR < 0.05
Supplemental Figure S1b Pathway analysis of correlation between general fatigue and metabolome of serum and saliva

## Slide 3
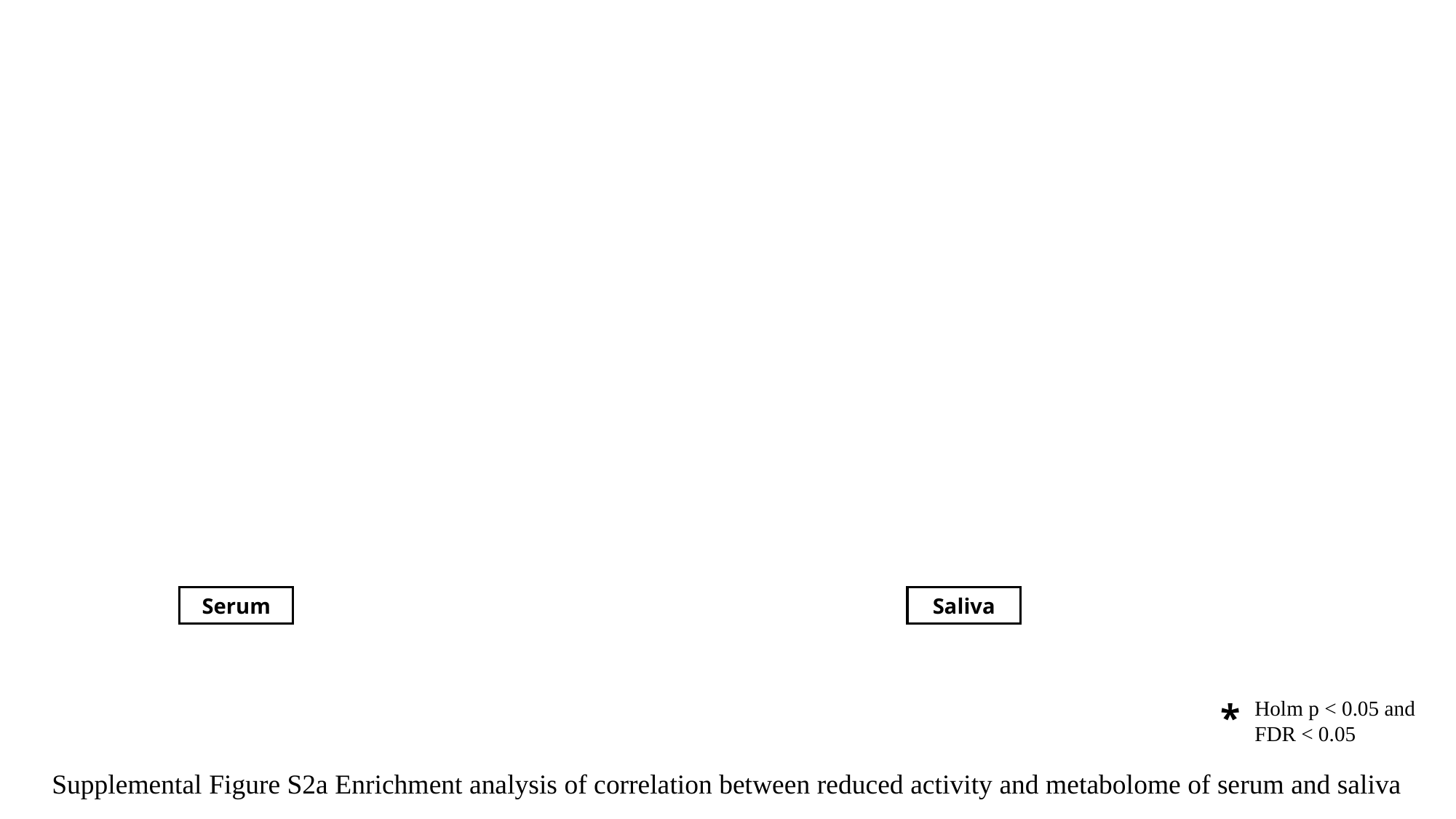

Serum
Saliva
*
Holm p < 0.05 and FDR < 0.05
Supplemental Figure S2a Enrichment analysis of correlation between reduced activity and metabolome of serum and saliva

## Slide 4
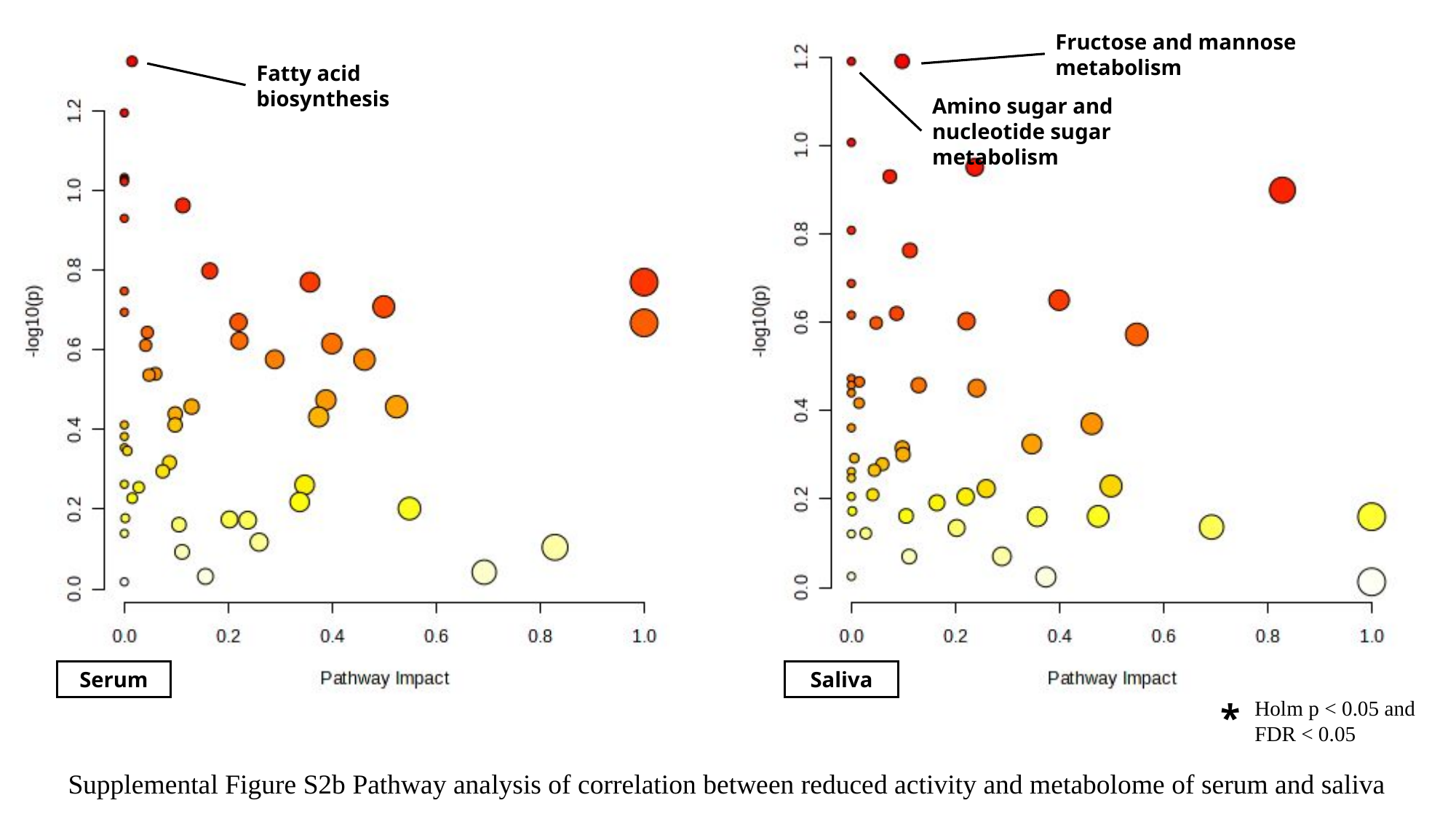

Fructose and mannose metabolism
Fatty acid biosynthesis
Amino sugar and nucleotide sugar metabolism
Serum
Saliva
*
Holm p < 0.05 and FDR < 0.05
Supplemental Figure S2b Pathway analysis of correlation between reduced activity and metabolome of serum and saliva

## Slide 5
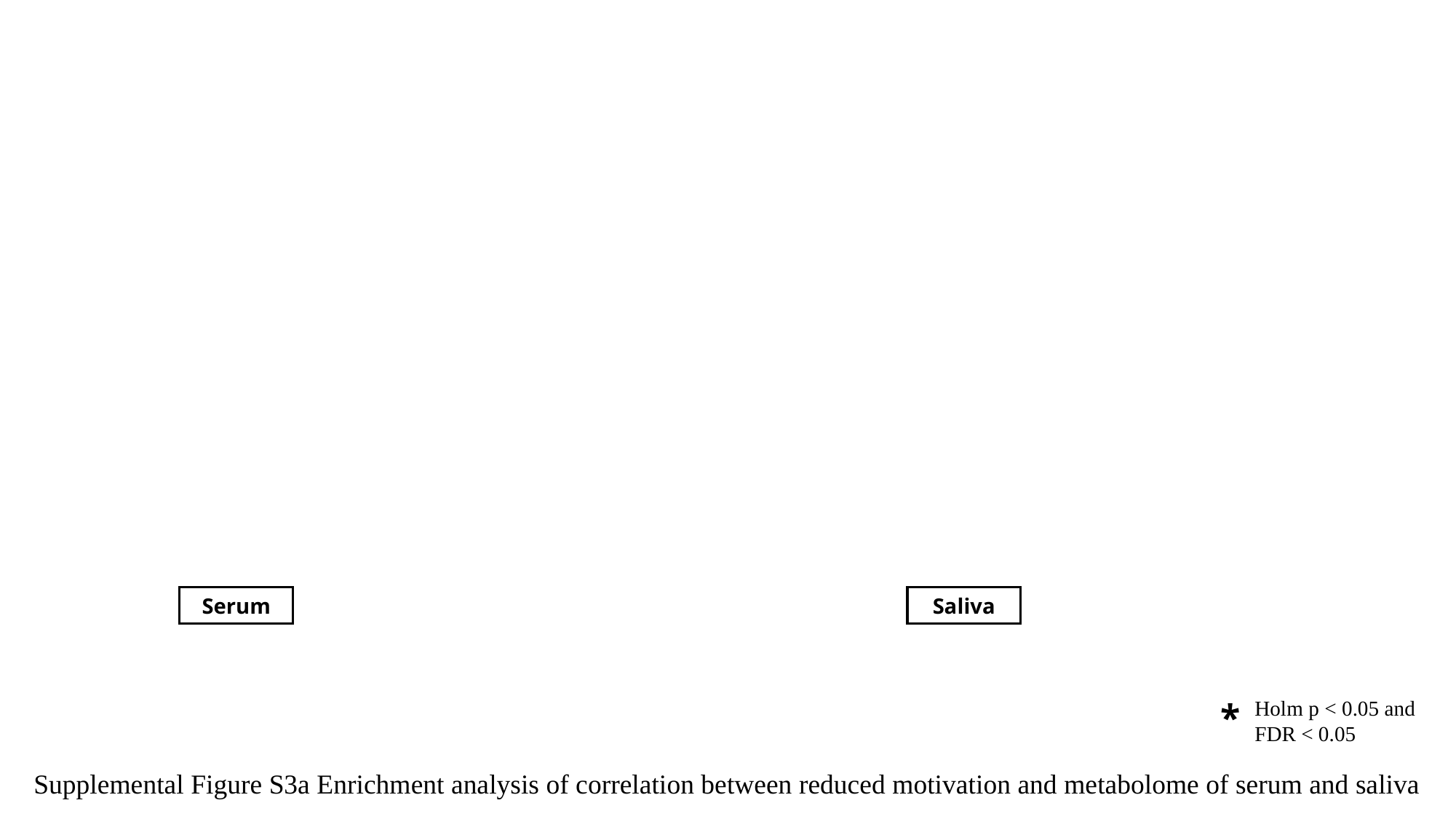

Serum
Saliva
*
Holm p < 0.05 and FDR < 0.05
Supplemental Figure S3a Enrichment analysis of correlation between reduced motivation and metabolome of serum and saliva

## Slide 6
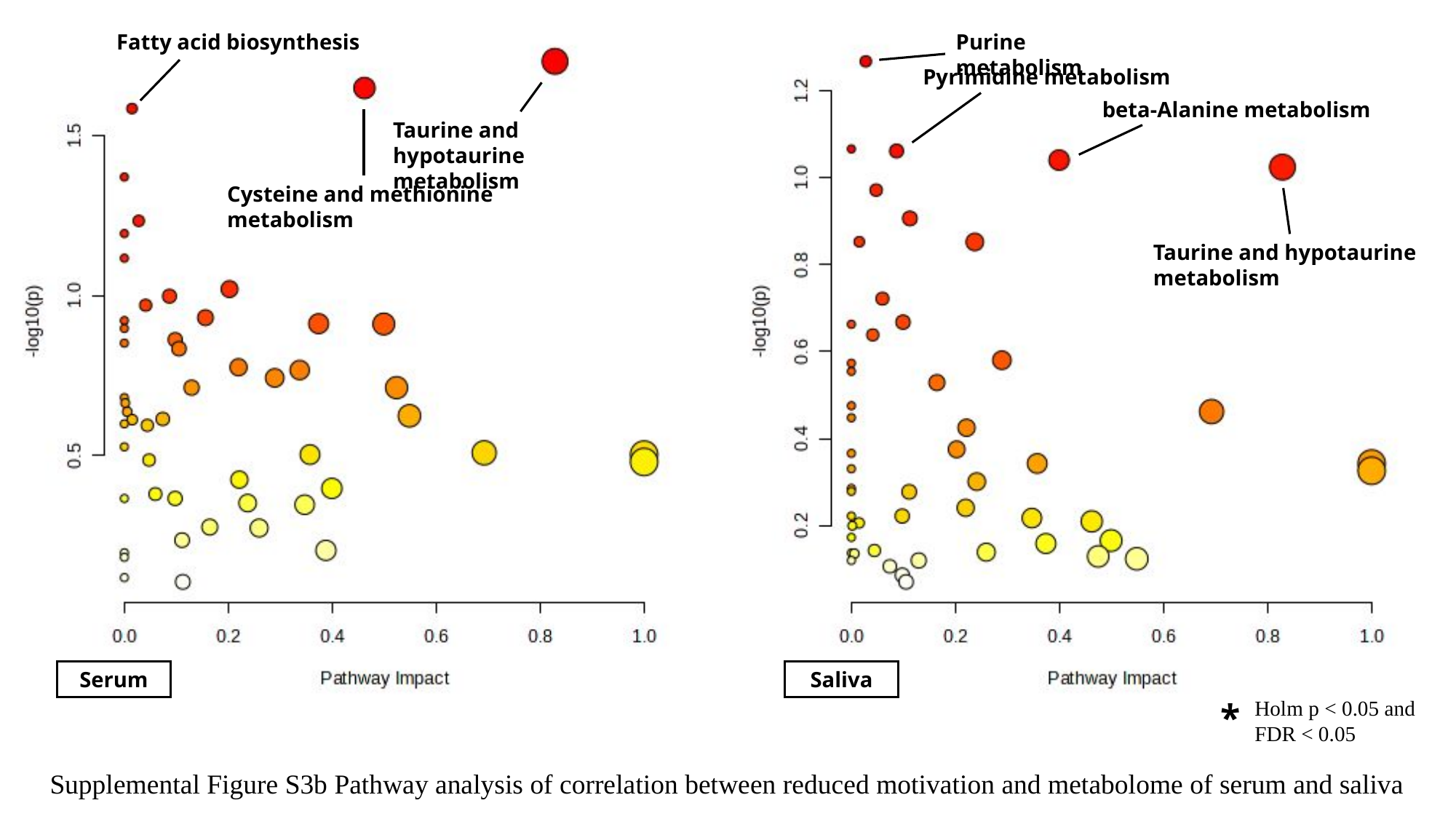

Fatty acid biosynthesis
Purine metabolism
Pyrimidine metabolism
beta-Alanine metabolism
Taurine and hypotaurine metabolism
Cysteine and methionine metabolism
Taurine and hypotaurine metabolism
Serum
Saliva
*
Holm p < 0.05 and FDR < 0.05
Supplemental Figure S3b Pathway analysis of correlation between reduced motivation and metabolome of serum and saliva

## Slide 7
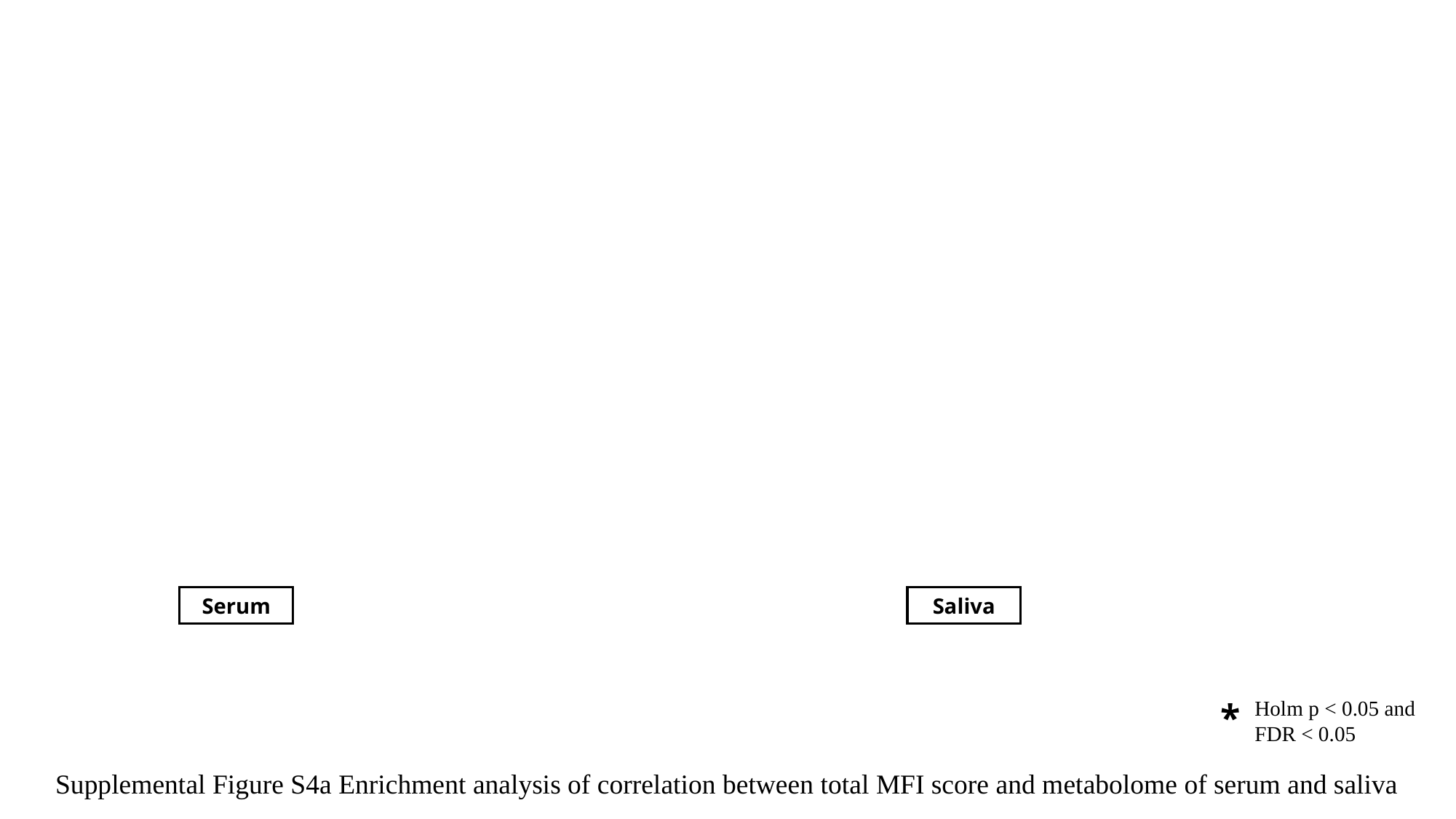

Serum
Saliva
*
Holm p < 0.05 and FDR < 0.05
Supplemental Figure S4a Enrichment analysis of correlation between total MFI score and metabolome of serum and saliva

## Slide 8
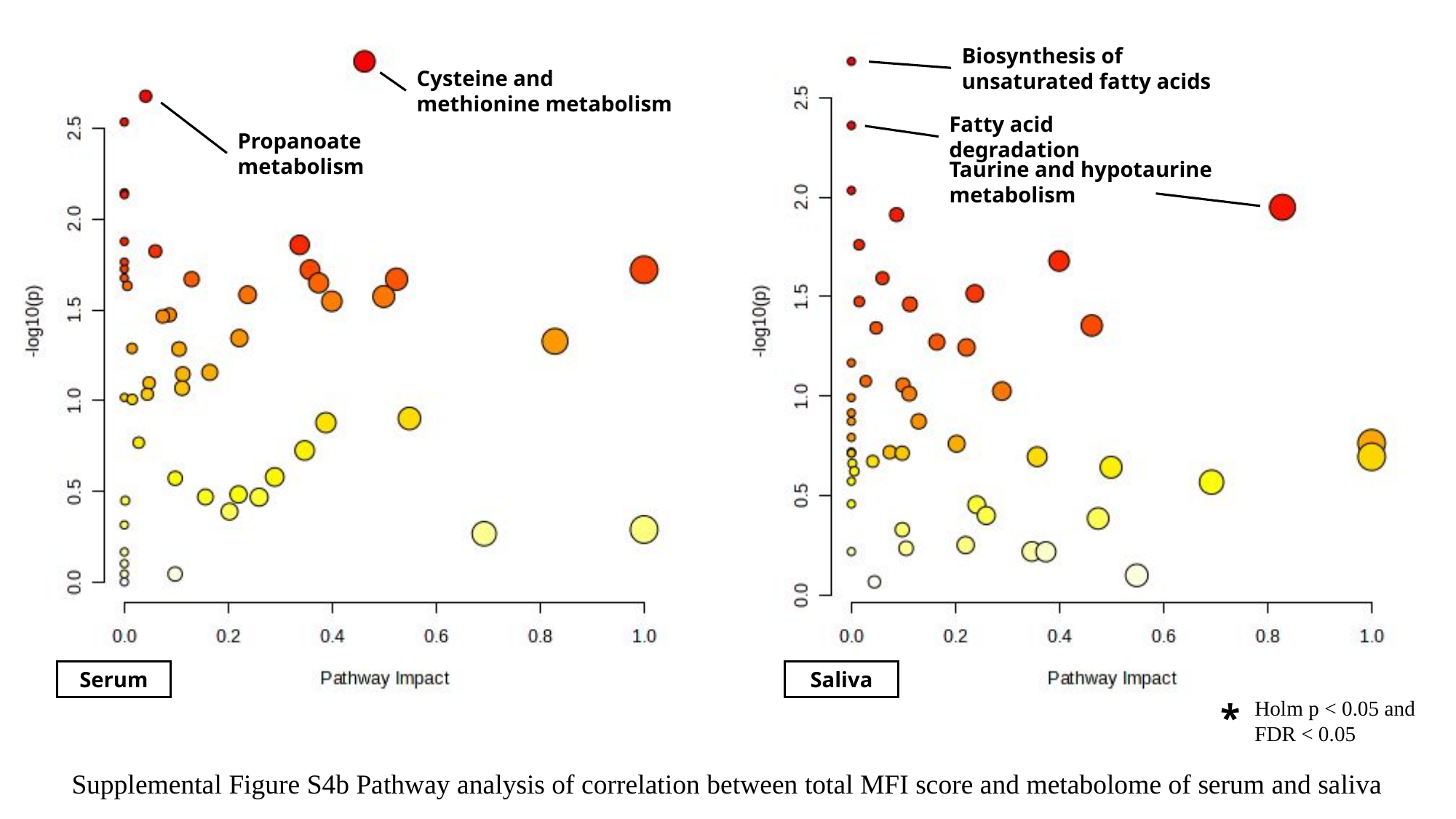

Biosynthesis of unsaturated fatty acids
Cysteine and methionine metabolism
Fatty acid degradation
Propanoate metabolism
Taurine and hypotaurine metabolism
Serum
Saliva
*
Holm p < 0.05 and FDR < 0.05
Supplemental Figure S4b Pathway analysis of correlation between total MFI score and metabolome of serum and saliva

## Slide 9
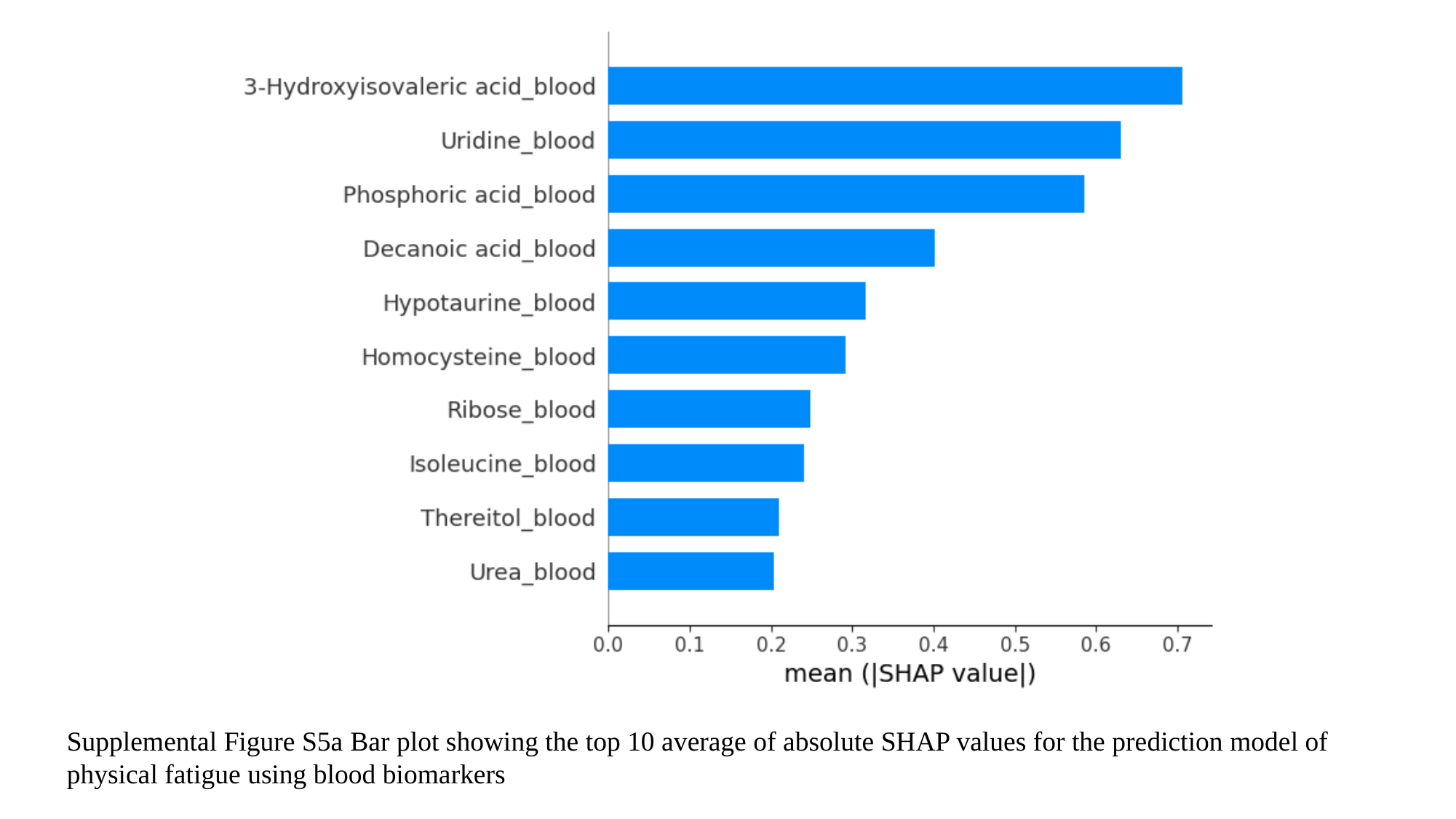

Supplemental Figure S5a Bar plot showing the top 10 average of absolute SHAP values for the prediction model of physical fatigue using blood biomarkers

## Slide 10
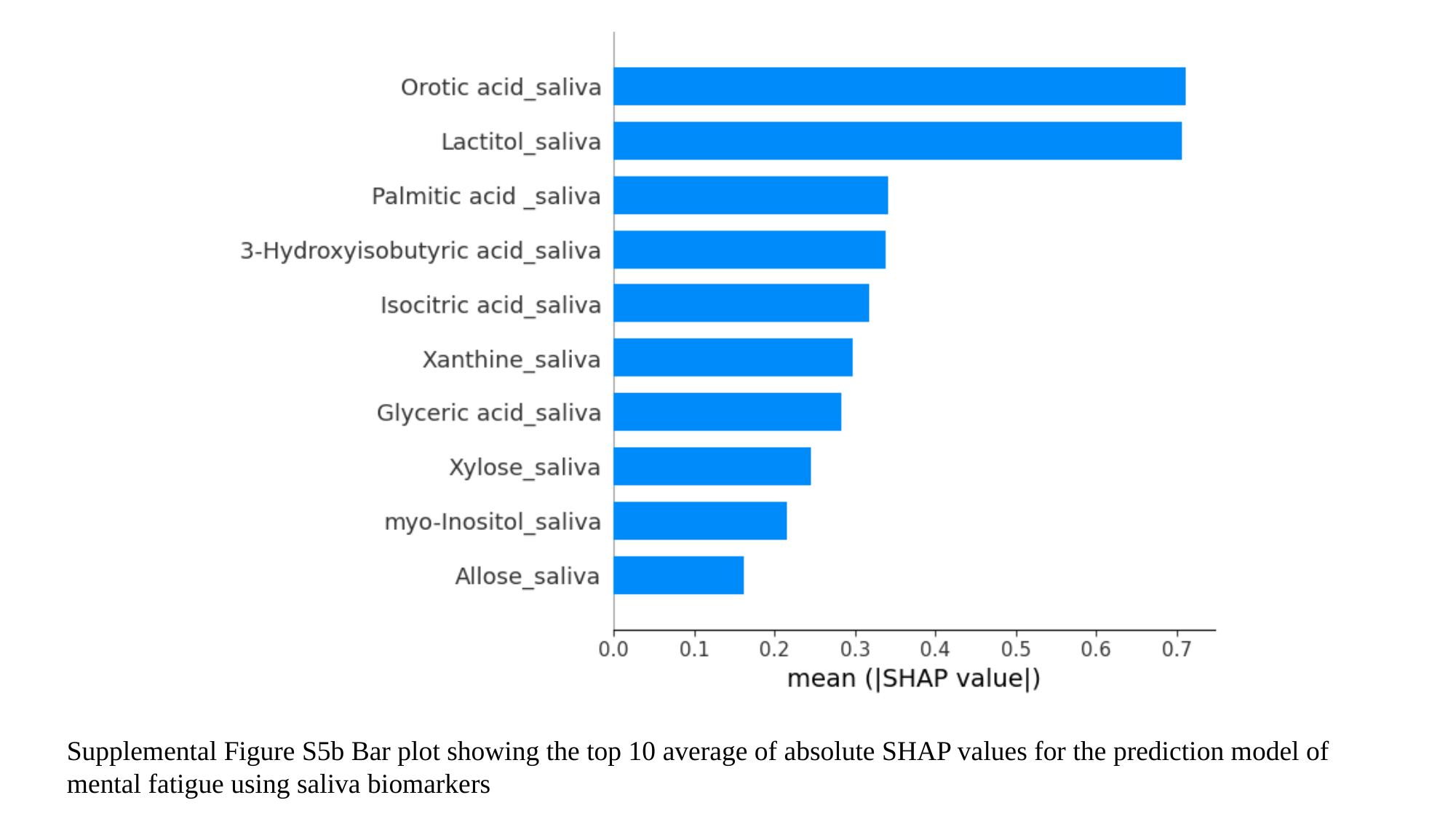

Supplemental Figure S5b Bar plot showing the top 10 average of absolute SHAP values for the prediction model of mental fatigue using saliva biomarkers
