# Supplementary material for: Visualizing fatigue mechanisms in non-communicable diseases: an integrative approach with multi-omics and machine learning
Source: BMC Med Inform Decis Mak. 2025 Jun 3;25:204. doi: 10.1186/s12911-025-03034-3 (PMC12135302; doi:10.1186/s12911-025-03034-3)
Supplement: Supplementary file 4 — Supplementary Material 4 [file 12911_2025_3034_MOESM4_ESM.docx]

**Supplemental Table S1 Correlations between alpha and beta diversity indices and fatigue dimensions.**

|  | Shannon index  r (p-value) | Simpson index  r (p-value) | Dimention 1  r (p-value) | Dimention 2  r (p-value) |
| --- | --- | --- | --- | --- |
| General fatigue | -0.137 (0.331) | -0.124 (0.380) | 0.123 (0.387) | 0.175 (0.216) |
| Physical fatigue | -0.166 (0.239) | -0.165 (0.243) | 0.095 (0.504) | 0.146 (0.301) |
| Mental fatigue | -0.076 (0.593) | -0.092 (0.518) | -0.041 (0.775) | -0.035 (0.804) |
| Reduced activity | 0.035 (0.806) | 0.027 (0.852) | 0.060 (0.674) | -0.092 (0.517) |
| Reduced motivation | -0.021 (0.880) | 0.014 (0.920) | 0.269 (0.053) | -0.055 (0.699) |
| Total MFI score | -0.092 (0.516) | -0.086 (0.545) | 0.121 (0.394) | 0.043 (0.763) |

This table presents the Pearson correlation coefficients (r) and corresponding p-values for associations between alpha diversity (Shannon and Simpson indices) and beta diversity (MDS-derived dimensions 1 and 2) with different dimensions of fatigue, including general fatigue, physical fatigue, mental fatigue, reduced activity, reduced motivation, and the total MFI score. No significant correlations were observed. Abbreviations: MFI, Multidimensional Fatigue Inventory; MDS, Multidimensional Scaling.

**Supplemental Table S2 Comparison of prediction model performance**

| Fatigue | Data | Model | ROC-AUC | Accuracy | F1 score |
| --- | --- | --- | --- | --- | --- |
| Physical | all data | Logistic regression | 0.585 | 0.635 | 0.689 |
| Physical | all data | LightGBM | 0.574 | 0.558 | 0.635 |
| Physical | all data | RUSBoostClassifier | 0.527 | 0.673 | 0.730 |
| Physical | salivary biomarkers | Logistic regression | 0.585 | 0.635 | 0.689 |
| Physical | salivary biomarkers | LightGBM | 0.574 | 0.558 | 0.635 |
| Physical | salivary biomarkers | RUSBoostClassifier | 0.527 | 0.673 | 0.730 |
| Physical | blood biomarkers | Logistic regression | 0.512 | 0.519 | 0.576 |
| Physical | blood biomarkers | LightGBM | 0.632 | 0.596 | 0.644 |
| Physical | blood biomarkers | RUSBoostClassifier | 0.473 | 0.500 | 0.536 |
| Mental | all data | Logistic regression | 0.567 | 0.558 | 0.623 |
| Mental | all data | LightGBM | 0.674 | 0.635 | 0.667 |
| Mental | all data | RUSBoostClassifier | 0.471 | 0.538 | 0.613 |
| Mental | salivary biomarkers | Logistic regression | 0.567 | 0.558 | 0.623 |
| Mental | salivary biomarkers | LightGBM | 0.674 | 0.635 | 0.667 |
| Mental | salivary biomarkers | RUSBoostClassifier | 0.471 | 0.538 | 0.613 |
| Mental | blood biomarkers | Logistic regression | 0.536 | 0.500 | 0.567 |
| Mental | blood biomarkers | LightGBM | 0.547 | 0.596 | 0.667 |
| Mental | blood biomarkers | RUSBoostClassifier | 0.485 | 0.519 | 0.590 |

“Fatigue”, “Data”, and “Model” indicate prediction target, input type, type of prediction models respectively. Model performance was evaluated using receiver operating characteristic area under the curve (ROC-AUC), accuracy, and the F1 score.
